# Endothelial cells are not productively infected by SARS‐CoV‐2

**DOI:** 10.1002/cti2.1350

**Published:** 2021-10-24

**Authors:** Lilian Schimmel, Keng Yih Chew, Claudia J Stocks, Teodor E Yordanov, Patricia Essebier, Arutha Kulasinghe, James Monkman, Anna Flavia Ribeiro dos Santos Miggiolaro, Caroline Cooper, Lucia de Noronha, Kate Schroder, Anne Karine Lagendijk, Larisa I Labzin, Kirsty R Short, Emma J Gordon

**Affiliations:** ^1^ Institute for Molecular Bioscience, Division of Cell and Developmental Biology The University of Queensland Brisbane QLD Australia; ^2^ School of Chemistry and Molecular Biosciences The University of Queensland Brisbane QLD Australia; ^3^ Institute for Molecular Bioscience, IMB Centre for Inflammation and Disease Research The University of Queensland Brisbane QLD Australia; ^4^ The University of Queensland Diamantina Institute The University of Queensland Brisbane QLD Australia; ^5^ School of Biomedical Science, Faculty of Health Queensland University of Technology Brisbane QLD Australia; ^6^ Postgraduate Program of Health Sciences School of Medicine Hospital Marcelino Champagnat ‐ Pontifícia Universidade Católica do Paraná (PUCPR) Curitiba Brazil; ^7^ Pathology Queensland Princess Alexandra Hospital Brisbane QLD Australia; ^8^ Faculty of Medicine The University of Queensland Brisbane QLD Australia; ^9^ School of Medicine & Center of Education, Research and Innovation Hospital Marcelino Champagnat ‐ Pontifícia Universidade Católica do Paraná (PUCPR) Curitiba Brazil

**Keywords:** blood vessels, COVID‐19, endothelial cells, inflammation, SARS‐CoV‐2

## Abstract

**Objectives:**

Thrombotic and microvascular complications are frequently seen in deceased COVID‐19 patients. However, whether this is caused by direct viral infection of the endothelium or inflammation‐induced endothelial activation remains highly contentious.

**Methods:**

Here, we use patient autopsy samples, primary human endothelial cells and an *in vitro* model of the pulmonary epithelial–endothelial cell barrier.

**Results:**

We show that primary human endothelial cells express very low levels of the SARS‐CoV‐2 receptor ACE2 and the protease TMPRSS2, which blocks their capacity for productive viral infection, and limits their capacity to produce infectious virus. Accordingly, endothelial cells can only be infected when they overexpress ACE2, or are exposed to very high concentrations of SARS‐CoV‐2. We also show that SARS‐CoV‐2 does not infect endothelial cells in 3D vessels under flow conditions. We further demonstrate that in a co‐culture model endothelial cells are not infected with SARS‐CoV‐2. Endothelial cells do however sense and respond to infection in the adjacent epithelial cells, increasing ICAM‐1 expression and releasing pro‐inflammatory cytokines.

**Conclusions:**

Taken together, these data suggest that *in vivo,* endothelial cells are unlikely to be infected with SARS‐CoV‐2 and that infection may only occur if the adjacent pulmonary epithelium is denuded (basolateral infection) or a high viral load is present in the blood (apical infection). In such a scenario, whilst SARS‐CoV‐2 infection of the endothelium can occur, it does not contribute to viral amplification. However, endothelial cells may still play a key role in SARS‐CoV‐2 pathogenesis by sensing adjacent infection and mounting a pro‐inflammatory response to SARS‐CoV‐2.

## Graphical abstract

We show that endothelial cells are not productively infected with SARS‐CoV‐2 but mount a pro‐inflammatory response to the virus.

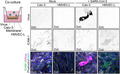

## INTRODUCTION

SARS‐CoV‐2 causes diverse clinical syndromes, ranging from asymptomatic infection to fatal disease. Of patients hospitalised with COVID‐19 (the clinical manifestation of SARS‐CoV‐2 infection), approximately 30% of individuals go on to develop severe disease associated with progressive lung damage.^1^ This is associated with a breakdown of the vascular barrier, oedema, endotheliitis, thrombosis and inflammatory cell infiltration.^1^, ^2^ Many studies have identified thrombotic and microvascular complications in deceased patients, suggesting that vascular pathology is a major driver of severe disease.^3^, ^4^, ^5^ Other major pathological events include arterial and venous thromboembolism, kidney disease and neurological disorders,^6^, ^7^ suggesting that SARS‐CoV‐2 activates the vasculature throughout the body, potentially resulting in multi‐organ failure. However, whether this is caused by direct viral infection of the endothelium or inflammation‐induced endothelial activation remains highly contentious.

SARS‐CoV‐2 cellular uptake is mediated by binding of the spike glycoprotein to ACE2 (angiotensin‐converting enzyme 2) and NRP1 (Neuropilin‐1) at the cell surface.^8^, ^9^, ^10^, ^11^ Host surface proteases such as TMPRSS2 cleave full‐length spike protein (S0) at its S2' site. Cleavage at the S2' site facilitates the fusion of viral and cellular membranes to deliver the viral RNA into the cytosol.^1^, ^12^ In cells with low expression of TMPRSS2, alternative routes of virus uptake involving the endo‐lysosomal pathway and cathepsins are also reported.^13^ Numerous studies suggest that endothelial cells express ACE2,^3^, ^14^, ^15^, ^16^, ^17^, ^18^ Neuropilin receptors^19^, ^20^, ^21^, ^22^, ^23^ and TMPRSS2,^24^ suggesting that viral infection of endothelial cells is theoretically possible. However, others have suggested that ACE2 is instead highly expressed in microvascular pericytes^15^, ^25^, ^26^ and that pericyte injury in response to viral infection may induce endothelial dysfunction.

It remains unclear whether SARS‐CoV‐2 can directly infect the endothelium.^27^, ^28^ Autopsy studies of deceased COVID‐19 patients have suggested the presence of viral particles in the vascular beds of different organs^3^, ^29^, ^30^ and that SARS‐CoV‐2 RNA is enriched in the pulmonary endothelial cells.^31^ Other studies have shown that SARS‐CoV‐2 infects cultured endothelial cells^32^, ^33^ and human blood vessel organoids.^34^ In contrast to such reports, other studies have failed to find evidence of endothelial infection in deceased COVID‐19 patients^28^ and questioned whether the presence of the SARS‐CoV‐2 spike protein was sufficient for identifying virally infected cells in previous studies.^26^, ^35^, ^36^ Animal models of SARS‐CoV‐2 infection have also failed to identify any obvious signs of endothelial infection,^37^, ^38^ despite clear endothelial dysfunction and thrombosis.^39^

When and how endothelial cells may be exposed to SARS‐CoV‐2 *in vivo* also remains unclear. As a respiratory virus, SARS‐CoV‐2 encounters pulmonary epithelial cells prior to any interaction with the endothelium. SARS‐CoV‐2 infects pulmonary epithelial cells apically and new viral particles are also released apically into the lumen of the lung.^40^ It is possible that the infected epithelial layer loses barrier function, allowing endothelial infection from the basolateral side of epithelia that is adjacent to the alveolar endothelium. Alternatively, SARS‐CoV‐2 may infect endothelial cells apically via the blood, although viraemia appears to be rare in COVID‐19 patients.^41^ The question therefore remains whether endothelial dysfunction in COVID‐19 results from direct viral infection of the endothelium.

Here, using patient autopsy samples and *in vitro* models, we show that whilst SARS‐CoV‐2 can enter endothelial cells if high viral titres are present, this infection is not productive. Rather, in response to direct or indirect viral exposure, endothelial cells mount a pro‐inflammatory response characterised by increased expression of ICAM‐1 and secretion of CXCL10 and IL‐6. Taken together, our results provide clarification on the intensely debated topic of endothelial infection by SARS‐CoV‐2, to reveal that the endotheliopathy and thrombocytopathy in patients with severe COVID‐19 are likely to be a result of the inflammatory response, rather than direct viral infection.

## RESULTS

### Endothelial cell infection is not readily detected in deceased COVID‐19 patients

To determine the prevalence of *in vivo* SARS‐CoV‐2 endothelial cell infection, autopsy lung sections were obtained from 10 deceased COVID‐19 patients and probed for SARS‐CoV‐2 spike mRNA using RNAscope. Two of the 10 patients were positive for SARS‐CoV‐2 RNA in the lungs. In these individuals, spike mRNA could not be detected in pulmonary endothelial cells (endothelial cells defined by H & E staining; Supplementary figure 1). These data suggest that the endothelium is not commonly the *in vivo* primary site of viral replication in COVID‐19 patients (patient data are shown in Supplementary table 1).

### Primary endothelial cells express low levels of ACE2 and TMPRSS2

**Figure 1:**
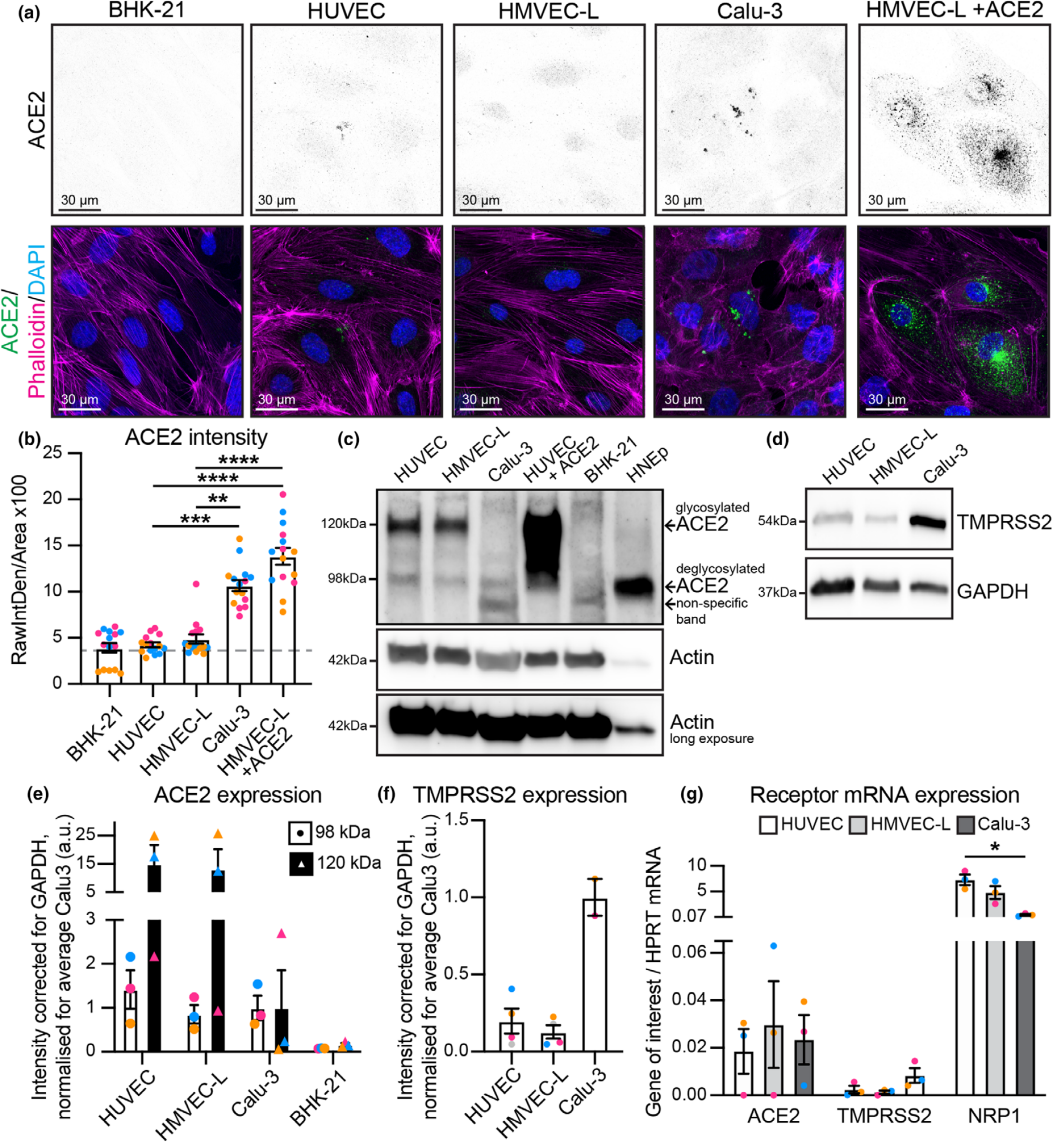
Endothelial cells express low levels of ACE2 and TMPRSS2 receptors. **(a)** Representative immunofluorescence images of BHK‐21, HUVEC, HMVEC‐L, Calu‐3 and HMVEC‐L + ACE2 overexpression cells stained for ACE2 (shown as single channel in top panel) (green), Phalloidin (magenta) and DAPI (blue). Scale bar 30 µm. **(b)** Quantification of ACE2 staining intensity, *n* = 15 images; 5 images per independent experiment, 3 independent experiments. **(c)** Western blot analysis showing both glycosylated (∼120 kDa) and deglycosylated (∼98 kDa) ACE2 protein. **(d)** Western blot analysis showing TMPRSS2 protein. **(e)** Quantification of protein levels for glycosylated ACE2 (∼120 kDa) and deglycosylated ACE2 (∼98 kDa) in HUVEC, HMVEC‐L, Calu‐3 and BHK‐21. *n* = 3 independent experiments. **(f)** Quantification of protein levels for TMPRSS2 in HUVEC, HMVEC‐L and Calu‐3. *n* = 4 (HUVEC and HMVEC‐L), *n* = 2 (Calu‐3) independent experiments. **(g)** qPCR shows presence of mRNA for ACE2, TMPRSS2 and NRP1 in HUVEC, HMVEC‐L and Calu‐3 cells. *n* = 3 independent experiments. Data are presented as mean ± s.e.m. with individual data points indicated and colour coded per independent experimental replicate. Statistical significance was determined using the Kruskal–Wallis test between Calu‐3 and HMVEC‐L +ACE2 and all others **(b)** or between Calu‐3 and all others **(e, f, g)**. *P < 0.05, ****P < 0.0001.

To further investigate the contribution of endothelial cells to the pathogenesis of SARS‐CoV‐2, we established *in vitro* cultures of primary human umbilical vein endothelial cells (HUVECs) and human microvascular endothelial cells from the lung (HMVEC‐L). Multiple studies have reported that endothelial cells across different vascular beds express ACE2 and TMPRSS2, the host cell receptor and protease that are required for efficient cell infection by SARS‐CoV‐2.^11^, ^12^, ^42^ The expression of ACE2 protein detected by immunofluorescence in HUVECs and HMVEC‐Ls (Figure 1a) was significantly reduced compared to immortalised epithelial cells known to be susceptible to SARS‐CoV‐2 infection (Calu‐3)^43^, ^44^ (Figure 1b). We also detected low expression of ACE2 and TMPRSS2 protein in HUVECs and HMVEC‐Ls by Western blot (Figure 1c–f). The specificity of our ACE2 antibody for both immunofluorescence and Western blot was confirmed by negative and positive controls, BHK‐21 cells (which are known to not express ACE2)^45^ and in HMVEC‐L cells stably overexpressing human ACE2. We primarily detected glycosylated ACE2 (∼120 kDa) and to a lesser extent deglycosylated ACE2 (∼98 kDa) in endothelial cells (HMVEC‐L, HUVEC),^46^ while overexpression of ACE2 in HUVEC increased expression of both the glycosylated (∼120 kDa) and non‐glycosylated (∼98 kDa) forms of ACE2. Interestingly, primary nasal epithelial cells and Calu‐3 cells primarily expressed non‐glycosylated ACE2 (Figure 1c and e), which fits with recent reports, suggesting that glycosylation of ACE2 may mask SARS‐CoV‐2 spike‐binding epitopes, and thereby prevent SARS‐CoV‐2 entry into target cells.^47^ Despite differences in ACE2 protein expression, endothelial cells expressed comparable ACE2 and TMPRSS2 mRNA to that observed in Calu‐3 (Figure 1g). Neuropilin‐1, which enhances viral entry through its interaction with the spike multibasic cleavage site,^8^, ^9^ was readily detected in endothelial cells by qPCR (Figure 1g) in line with established data.^20^ These data suggest that while vascular endothelial cells express the cellular machinery to permit SARS‐CoV‐2 infection, this is likely to be inefficient compared to epithelial cells because of weak receptor and protease expression in endothelial cells.

### Endothelial cells are not productively infected by SARS‐CoV‐2

SARS‐CoV‐2 may theoretically enter endothelial cells via the apical surface (should the virus enter the blood stream) or the basolateral surface (should the virus be present at the basolateral surface of the adjacent epithelial cells). We therefore assessed whether endothelial cells were susceptible to SARS‐CoV‐2 infection when exposed either apically (Supplementary figure 2a) or basolaterally (Supplementary figure 2b) to 6 × 10^4^ PFUs (estimated multiplicity of infection (MOI) = 1). Infectious virus was not detected in the supernatant of either HUVECs or HMVEC‐Ls at 24, 48 or 72 h post‐infection (Supplementary figure 2c). In contrast, Calu‐3 infection resulted in robust viral replication and release (Supplementary figure 2c). Similarly, we did not detect SARS‐CoV‐2 nucleocapsid protein in SARS‐CoV‐2‐treated endothelial cells by immunofluorescence (Supplementary figure 2d–g) or Western blot (Supplementary figure 2h and i), in contrast to Calu‐3 cells. Nor did we observe any detectable changes in endothelial cell morphology after viral exposure, as assessed by Phalloidin immunostaining (Supplementary figure 2d and f). Together, these data suggest that endothelial cells are not efficiently infected with SARS‐CoV‐2 *in vitro*.

**Figure 2:**
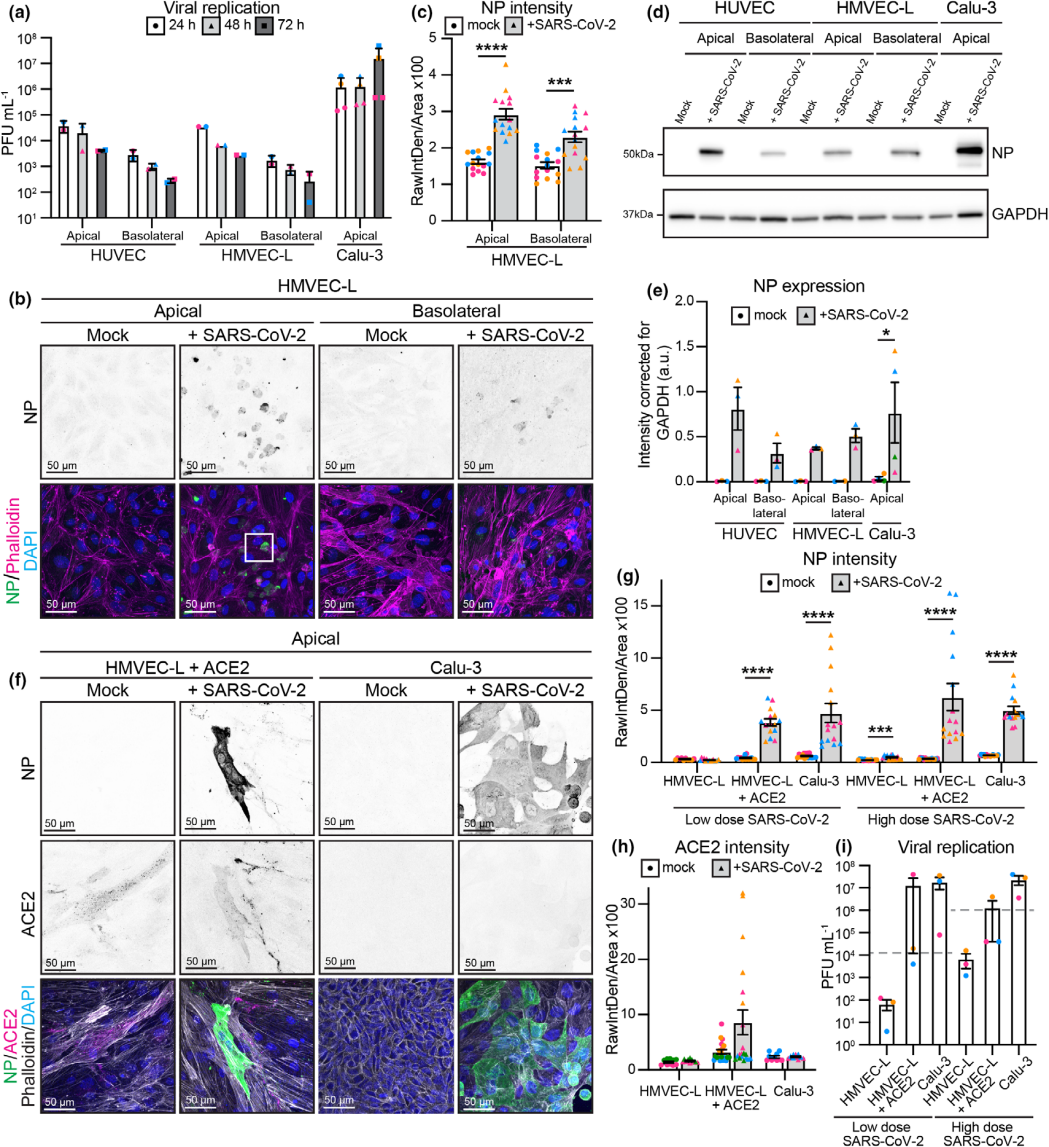
Endothelial cells can be infected with 2 × 10^6^ PFU of SARS‐CoV‐2, but infection is abortive. **(a)** Viral replication shown as number of PFU mL^−1^ of supernatant from SARS‐CoV‐2 infected HUVEC, HMVEC‐L and Calu‐3 cells at 24 h, 48 h and 72 h after infection. *n* = 2 (HUVEC and HMVEC‐L), *n* = 3 (Calu‐3) independent experiments. **(b)** Representative immunofluorescent images of HMVEC‐L stained for nucleocapsid protein (NP) (shown as single channel in top panel) (green), Phalloidin (magenta) and DAPI (blue) with mock or SARS‐CoV‐2 infection from either the apical or basolateral side of the cells at 72 h after infection. Scale bar 50 µm. The white box is enlarged in Figure 3. **(c)** Quantification of NP staining intensity in HMVEC‐L. *n* = 15 images from 3 independent experiments. **(d)** Western blot analysis showing NP protein levels in HUVEC, HMVEC‐L and Calu‐3 cells after 72 h of infection. **(e)** Quantification of NP protein levels in HUVEC, HMVEC‐L and Calu‐3. *n* = 3 independent experiments. **(f)** Representative immunofluorescent images of ACE2 overexpressing HMVEC‐L and control Calu‐3 cells stained for NP (shown as single channel in top panel) (green), ACE2 (shown as single channel in middle panel) (magenta), Phalloidin (grey) and DAPI (blue) with mock or SARS‐CoV‐2 infection from the apical side of the cells at 72 h after infection. Scale bar 50 µm. **(g)** Quantification of NP staining intensity in HMVEC‐L, HMVEC‐L + ACE2 and Calu‐3 with either low (6 × 10^4^) or high (2 × 10^6^) dose of SARS‐CoV‐2 infection. *n* = 15 images from 3 independent experiments. **(h)** Quantification of ACE2 staining intensity in HMVEC‐L, HMVEC‐L + ACE2 and Calu‐3. *n* = 10 (HMVEC‐L and Calu‐3), *n* = 20 (HMVEC‐L + ACE2) images from 2 independent experiments. **(i)** Viral replication shown as number of PFU mL^−1^ of supernatant from SARS‐CoV‐2 infected HMVEC‐L, HMVEC‐L + ACE2 and Calu‐3 cells at 72 h after infection. The grey dashed line indicates input level. *n* = 3 independent experiments. Data are presented as mean ± s.e.m. with individual data points indicated and colour coded per independent experimental replicate. Statistical significance was determined using the Kruskal–Wallis test between 24 h and other time points **(a)**, between HMVEC‐L and all other conditions **(i)** or the Mann–Whitney *U*‐test between mock and + SARS‐CoV‐2 **(c, e, g)**. *P < 0.05, ***P < 0.001, ****P < 0.0001.

We next established whether a higher viral titre could overcome the low levels of ACE2 and TMPRSS2 on endothelial cells to mediate productive infection. Accordingly, endothelial cells were infected either apically or basolaterally with 2 × 10^6^ PFUs of SARS‐CoV‐2 and viral replication was again assessed over time (estimated MOI = 30). Infectious virus did not increase over time in endothelial cells; rather viral titres decreased over time, consistent with the degradation of input virus (Figure 2a). Similarly, ACE2‐negative BHK‐21 cells showed no evidence of productive viral replication, as expected (Supplementary figure 3a). In contrast, productive viral replication was observed in virus‐susceptible Calu‐3 cells over time (Figure 2a). To determine whether endothelial cells were susceptible to viral entry, if not replication, we assessed viral nucleocapsid protein in HMVEC‐L (Figure 2b and c) and positive control Calu‐3 and negative control BHK‐21 cells (Supplementary figure 3d and e) by immunofluorescence, which was further validated by Western blot (Figure 2d and e, Supplementary figure 3b and c). Viral nucleocapsid protein expression was significantly increased after both apical and basolateral SARS‐CoV‐2 infection, revealing that SARS‐CoV‐2 can indeed enter endothelial cells *in vitro*, but this infection is abortive.

To determine whether low ACE2 expression was the only factor limiting productive SARS‐CoV‐2 replication in endothelial cells, we stably overexpressed human ACE2 in HMVEC‐L cells (Figure 1). We assessed viral entry by immunofluorescence for nucleocapsid protein in HMVEC‐L + ACE2 cells infected with 2 × 10^6^ PFUs of SARS‐CoV‐2 (Figure 2f and g, Supplementary figure 4b) or 6 × 10^4^ PFUs of SARS‐CoV‐2 (Supplementary figure 4a). We observed a significant increase in nucleocapsid protein intensity following infection in HMVEC‐L + ACE2, similar to that observed in Calu‐3 cells at low and high viral titres (Figure 2g). Elevated expression of ACE2 in virus‐infected cells was confirmed by immunofluorescence (Figure 2f–h). In contrast to HMVEC‐L with endogenous levels of ACE2, overexpression of ACE2 in HMVEC‐L resulted in active viral replication upon infection with either 2 × 10^6^ PFUs of SARS‐CoV‐2 or 6 × 10^4^ PFUs of SARS‐CoV‐2 (Figure 2i).

### Endothelial cells undergo apoptosis and are extruded

**Figure 3:**
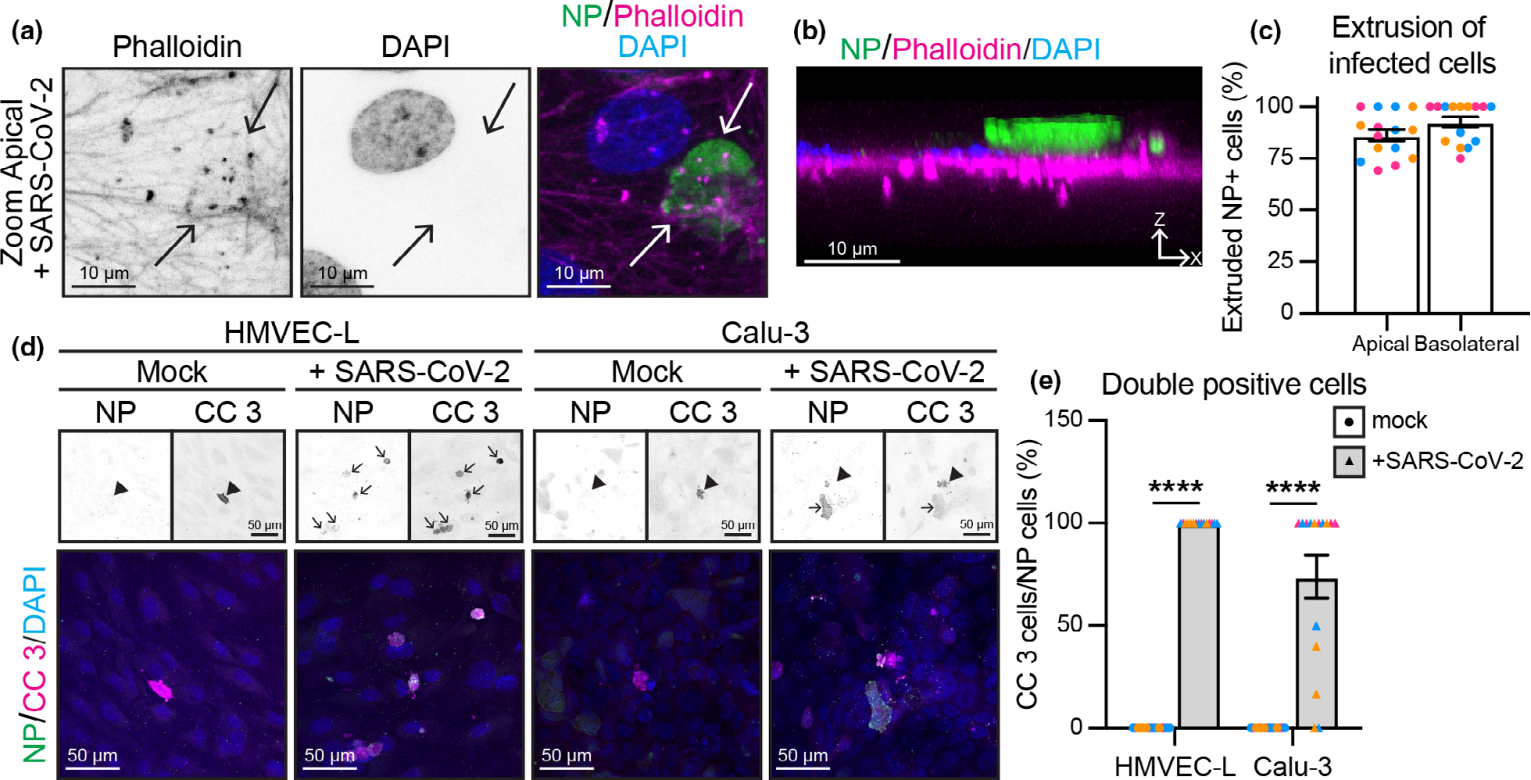
Endothelial cell infection results in apoptosis and cell extrusion. **(a)** Enlargement of the white box in Figure 2b shows Phalloidin and DAPI single channels and merged channel. Scale bar 10 µm. **(b)** XZ projection of merged image in (a), with apical side on top of image and basolateral side on the bottom. Scale bar 10 µm. **(c)** Quantification of % nucleocapsid protein (NP)‐positive cells that are extruded upon SARS‐CoV‐2 infection of HMVEC‐Ls. *n* = 15 images from 3 independent experiments. **(d)** Representative immunofluorescence images of HMVEC‐L and Calu‐3 stained for NP (shown as single channel in top left panel) (green), cleaved caspase 3 (CC 3) (shown as single channel in top right panel) (magenta) and DAPI (blue) with mock or SARS‐CoV‐2 infection from the apical side of the cells at 72 h after infection. Scale bar 50 µm. **(e)** Quantification of % NP‐positive cells that are positive for the apoptosis marker cleaved caspase 3. *n* = 15 images from 3 independent experiments. Data are presented as mean ± s.e.m. with individual data points indicated and colour coded per independent experimental replicate. Statistical significance was determined using the Mann–Whitney *U*‐test between mock and + SARS‐CoV‐2 **(e)**. ****P < 0.0001.

In endothelial cells with endogenous expression of ACE2, we observed that the majority of infected, nucleocapsid protein‐positive cells appeared to be extruding from the cell monolayer in an apical manner (Figure 3a–c). Such observations could not be accounted for by dead cells present in the viral inoculum, as challenged BHK‐21 cells did not extrude nucleocapsid‐positive cells (Supplementary figure 3d). Moreover, extruding endothelial cells were positive for cleaved caspase 3, indicating that SARS‐CoV‐2 entry into endothelial cells induces apoptosis (Figure 3d and e). These results demonstrate that while SARS‐CoV‐2 can enter endothelial cells at a high viral inoculum, this infection is not productive and induces apoptosis and resultant cell extrusion from the endothelial monolayer.

### Endothelial cells are not productively infected by SARS‐CoV‐2 in 3D vessels

**Figure 4:**
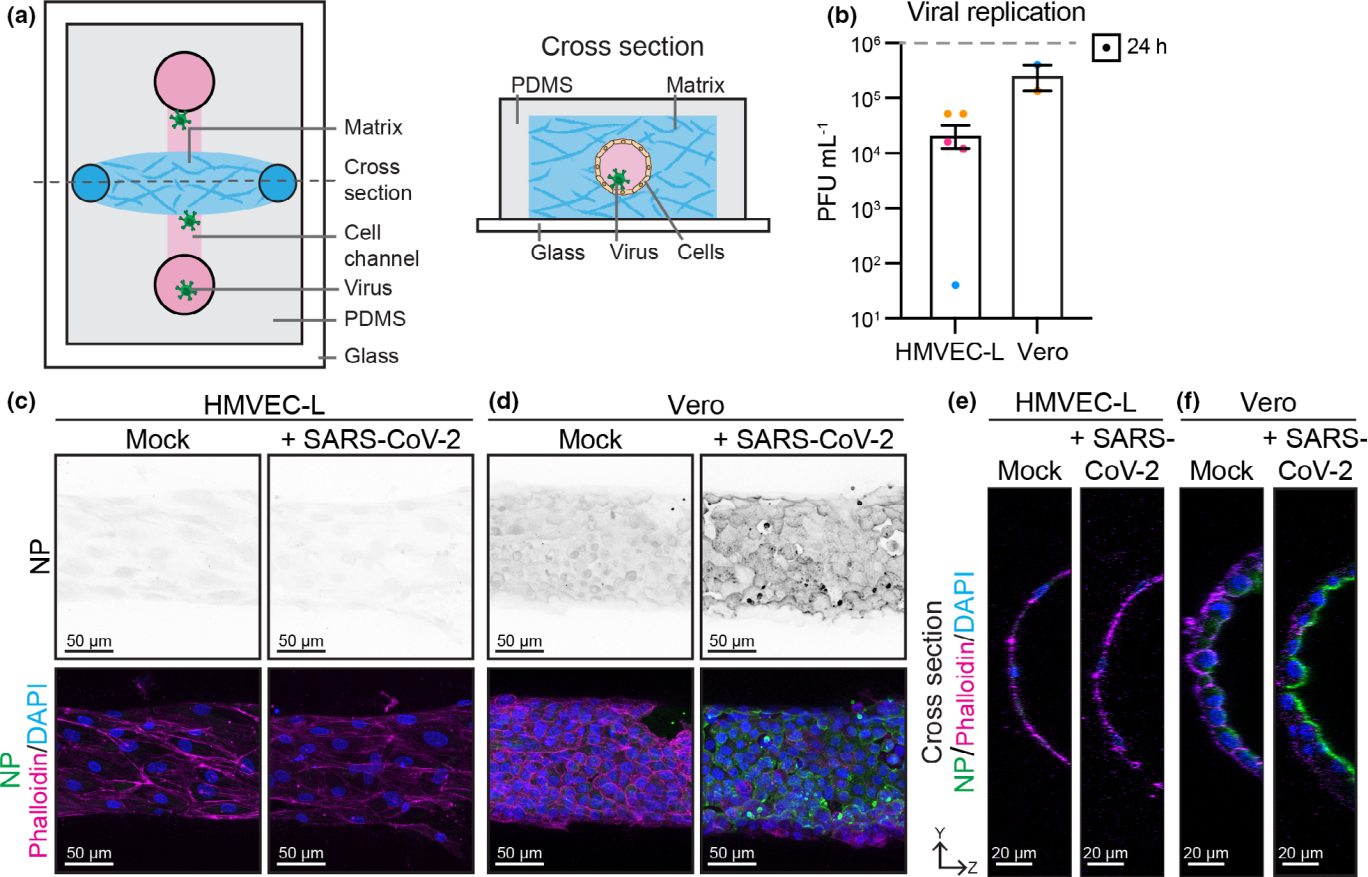
Endothelial cells are not infected with 2 × 10^6^ PFU of SARS‐CoV‐2 in 3D flow‐pressured tubes. **(a)** Schematic of microfabricated device used to culture 3D vessel tubes under oscillatory flow. The cross‐section shows the lumenised tube covered with endothelial cells surrounded by extracellular matrix. Virus can be flowed through the lumen of the tubes. **(b)** Viral replication shown as number of PFU mL^−1^ of supernatant from SARS‐CoV‐2‐infected HMVEC‐L and Vero cells at 24 h after infection. *n* = 3 (HMVEC‐L), *n* = 2 (Vero) independent experiments. Representative immunofluorescence images of **(c)** HMVEC‐L and **(d)** Vero stained for nucleocapsid protein (NP) (shown as single channel in top panel) (green), Phalloidin (magenta) and DAPI (blue) with mock or SARS‐CoV‐2 infection in the lumen of the tube at 24 h after infection. Scale bar 50 µm. YZ projection of HMVEC‐L (merged image in **c**) and **(f)** Vero (merged image in **d**), with luminal side towards the right of image and basolateral side towards the left of images. Scale bar 20 µm.

To determine whether endothelial cells can be infected in 3D vessels under flow, we cultured both HMVEC‐L and Vero cells in microfabricated tubes^48^ and added SARS‐CoV‐2 to the luminal surface (Figure 4a). After 24 h, Vero cells were readily infected and viral replication was observed, in contrast to HMVEC‐L which showed no increase in viral titre above input levels (Figure 4b). In agreement, viral nucleocapsid protein was not detected in HMVEC‐L tubes, whereas Vero cells displayed clear nucleocapsid protein staining at the luminal surface (Figure 4c–f). These data reveal that fluid flow over endothelial cells does not alter the endothelial cell susceptibility to SARS‐CoV‐2 infection.

### Endothelial cells mount a modest immune response to SARS‐CoV‐2

**Figure 5:**
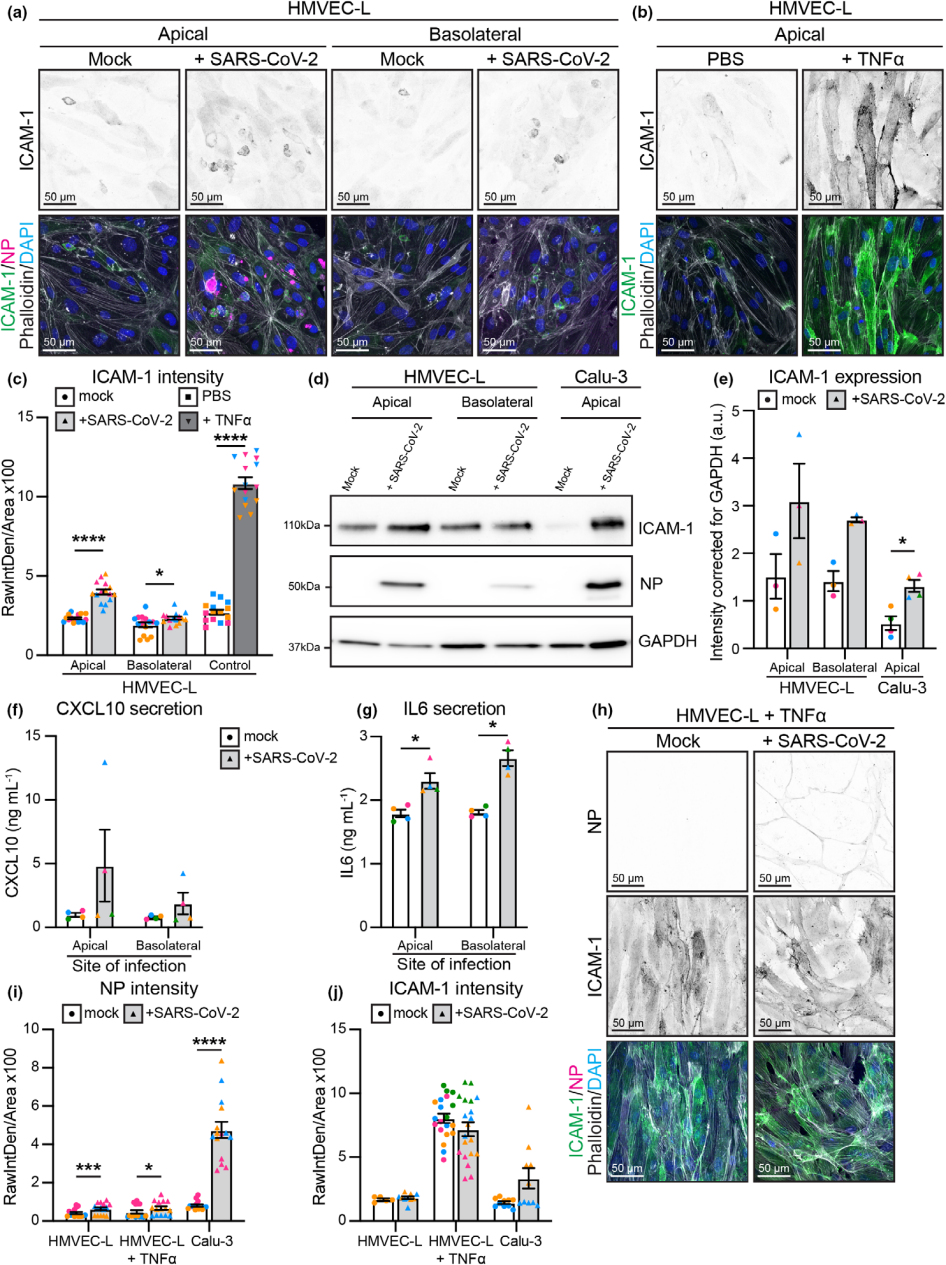
Endothelial cells mount an inflammatory response upon SARS‐CoV‐2 infection. Representative immunofluorescence images of HMVEC‐L stained for ICAM‐1 (shown as single channel in top panel) (green), NP (magenta), Phalloidin (grey) and DAPI (blue) with **(a)** mock or SARS‐CoV‐2 infection from either the apical or basolateral side of the cells at 72 h after infection, or with **(b)** control and TNFα treatment for 72 h. Scale bar 50 µm. **(c)** Quantification of ICAM‐1 staining intensity in HMVEC‐L. *n* = 15 images from 3 independent experiments. **(d)** Western blot analysis showing ICAM‐1 and nucleocapsid protein (NP) protein levels in HMVEC‐L and Calu‐3 cells after 72 h of infection. **(e)** Quantification of ICAM‐1 protein levels in HMVEC‐L and Calu‐3. *n* = 3 independent experiments. Measurement of cytokines with an AlphaLISA Immunoassay kit for **(f)** CXCL10 and **(g)** IL‐6 in the supernatant of HMVEC‐L with mock or SARS‐CoV‐2 infection from either the apical or basolateral side of the cells at 72 h after infection. *n* = 4 independent experiments. **(h)** Representative immunofluorescence images of HMVEC‐L pre‐treated for 16 h with TNFα stained for NP (shown as single channel in top panel) (magenta), ICAM‐1 (shown as single channel in middle panel) (green), Phalloidin (grey) and DAPI (blue) with mock or SARS‐CoV‐2 infection from the apical side of the cells at 72 h after infection. Scale bar 50 µm. **(i)** Quantification of NP staining intensity in HMVEC‐L, HMVEC‐L + TNFα and Calu‐3 with SARS‐CoV‐2 infection. *n* = 15 images from 3 independent experiments. **(j)** Quantification of ICAM‐1 staining intensity in HMVEC‐L, HMVEC‐L + TNFα and Calu‐3. *n* = 10 (HMVEC‐L and Calu‐3), *n* = 20 (HMVEC‐L + TNFα) images from 2 independent experiments. Data are presented as mean ± s.e.m. with individual data points indicated and colour coded per independent experimental replicate. Statistical significance was determined using the Mann–Whitney *U*‐test between mock and + SARS‐CoV‐2 **(c, e, f, g, i)**. *P < 0.05, ***P < 0.001, ****P < 0.0001.

As severe COVID‐19 disease is associated with an elevated cytokine response,^49^ we next sought to assess whether the abortive SARS‐CoV‐2 infection observed in endothelial cells induces a pro‐inflammatory response. We assessed expression of the leukocyte adhesion molecule, ICAM‐1, which is well established to be induced under inflammatory conditions in the endothelium.^50^ Immunofluorescent staining showed that HMVEC‐L exposed to SARS‐CoV‐2 significantly increased ICAM‐1 expression, regardless of whether cells were infected apically or basally (Figure 5a–c). A notable exception was for HMVEC‐L cells that stained positive for nucleocapsid protein; these appeared to be apoptotic and extruded from the monolayer. Mock infected cells demonstrated moderate basal ICAM‐1 expression, which is likely due to cell maintenance for 72 h without a media change (as seen in PBS controls, Figure 5b). However, ICAM‐1 induction was not as robust as when HMVEC‐L were exposed to the established inflammatory cytokine TNFα (Figure 5b and c). Western blot analysis confirmed that HMVEC‐L exposed to SARS‐CoV‐2 displayed a trending increase in ICAM‐1 expression (Figure 5d and e). In addition to upregulating inflammatory adhesion molecules, we assessed whether endothelial cells can be a source of pro‐inflammatory cytokines during SARS‐CoV‐2 infection. As IL‐6 and CXCL10 are elevated in patients with severe COVID‐19,^51^ we analysed whether endothelial cells released these pro‐inflammatory cytokines when infected either apically or basolaterally with SARS‐CoV‐2 for 72 h. HMVEC‐Ls released modest levels of CXCL10 (Figure 5f) and IL‐6 (Figure 5g). While these modest levels of cytokine release from endothelial cells upon SARS‐CoV‐2 infection are lower than CXCL10 or IL‐6 induced in response to high concentrations of recombinant TNFα or IFNß (Supplementary figure 5d and e), these data show that endothelial cells express high basal levels of IL‐6 and that both cytokines are increased during infection.

In order to determine whether inflamed endothelial cells are more prone to SARS‐CoV‐2 entry and replication, we pre‐treated HMVEC‐L with TNFα for 16 h before viral exposure and assessed viral entry by immunofluorescence for nucleocapsid protein. TNFα pre‐treatment of HMVEC‐L did not lead to further increase in nucleocapsid protein‐positive cells compared to untreated HMVEC‐L after infection with 2 × 10^6^ PFUs of SARS‐CoV‐2 (Figure 5h and i), despite increased ICAM‐1 intensity levels demonstrating TNFα‐driven HMVEC‐L cell activation (Figure 5j). Moreover, TNFα pre‐treatment of HMVEC‐L did not result in increased nucleocapsid protein‐positive cells upon infection with 6 × 10^4^ PFUs of SARS‐CoV‐2 (Supplementary figure 5a and b). Accordingly, we observed no increase in viral replication by plaque assay in TNFα pre‐treated HMVEC‐L, infected with either 2 × 10^6^ PFUs or 6 × 10^4^ PFUs of SARS‐CoV‐2 (Supplementary figure 5c).

Taken together, these results show that endothelial cells do not support a productive SARS‐CoV‐2 infection but still mount a modest pro‐inflammatory response to the virus. Furthermore, inflamed endothelial cells are not more susceptible to SARS‐CoV‐2 infection or productive SARS‐CoV‐2 viral replication.

### Infection of epithelial–endothelial co‐cultures elicits an immune response in endothelial cells

**Figure 6:**
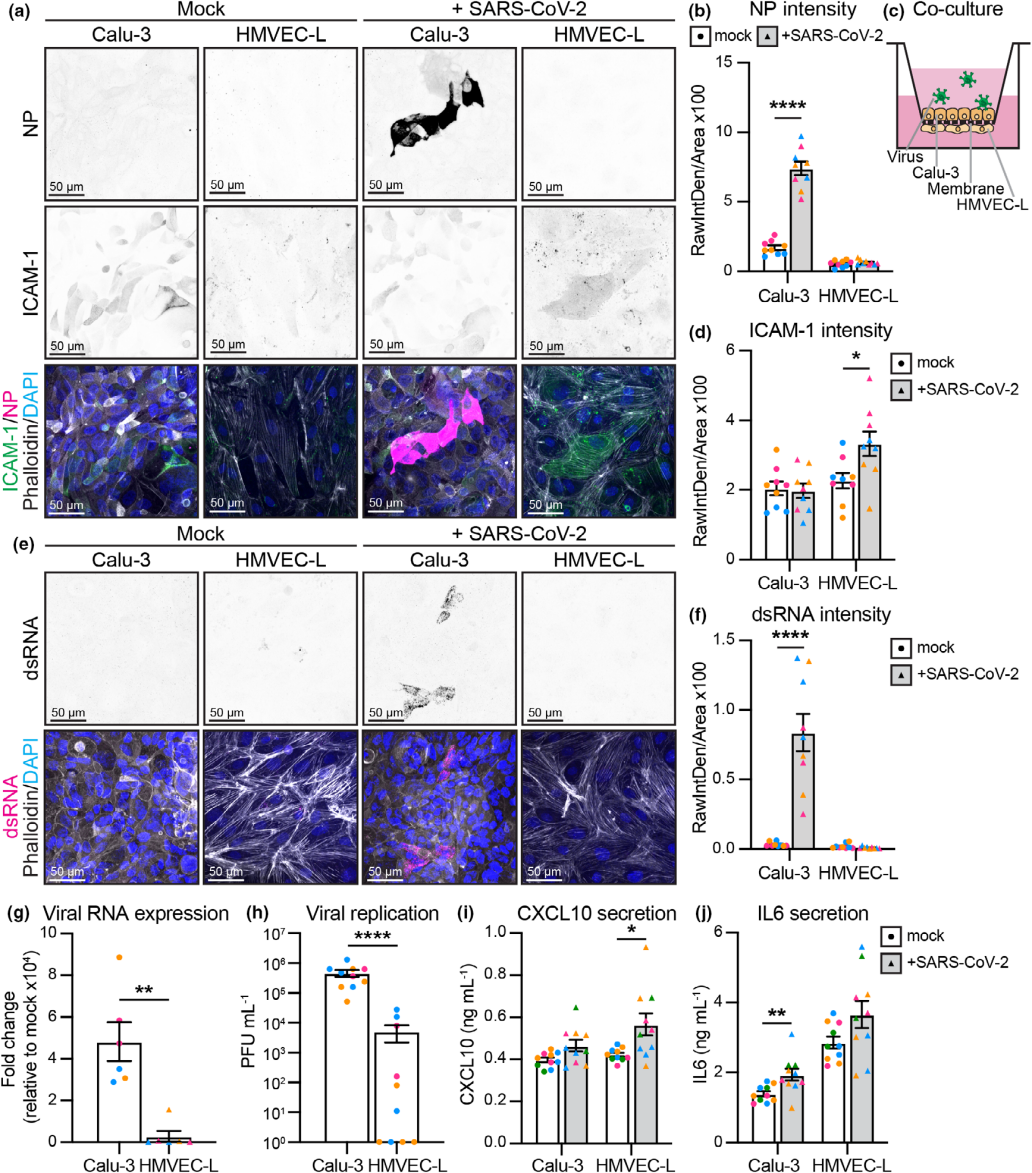
Co‐culture shows SARS‐CoV‐2 infection of Calu‐3 and inflammation of HMVEC‐L. **(a)** Representative immunofluorescence images of co‐cultured Calu‐3 and HMVEC‐L stained for nucleocapsid protein (NP) (shown as single channel in top panel) (magenta), ICAM‐1 (shown as single channel in middle panel) (green), Phalloidin (grey) and DAPI (blue) with mock or SARS‐CoV‐2 infection at 72 h after infection (2 × 10^6^ PFU). Scale bar 50 µm. **(b)** Quantification of NP staining intensity in Calu‐3 and HMVEC‐L. *n* = 9 images from 3 independent experiments. **(c)** Schematic of Calu‐3 and HMVEC‐L co‐cultures on transwell membranes. **(d)** Quantification of ICAM‐1 staining intensity in Calu‐3 and HMVEC‐L. *n* = 9 images from 3 independent experiments. **(e)** Representative immunofluorescence images of co‐cultured Calu‐3 and HMVEC‐L stained for dsRNA (shown as single channel in top panel) (magenta), Phalloidin (grey) and DAPI (blue) with mock or SARS‐CoV‐2 infection at 72 h after infection. Scale bar 50 µm. **(f)** Quantification of dsRNA staining intensity in Calu‐3 and HMVEC‐L. *n* = 9 images from 3 independent experiments. **(g)** qPCR shows presence of viral RNA (MPRO) in SARS‐CoV‐2 infected Calu‐3 and HMVEC‐L co‐cultured cells, represented as fold change relative to mock infection at 72 h after infection. *n* = 3 independent experiments. **(h)** Viral replication shown as number of PFU mL^−1^ of supernatant from SARS‐CoV‐2 infected Calu‐3 and HMVEC‐L co‐cultured cells at 72 h after infection. *n* = 3 independent experiments. Measurement of cytokines with an AlphaLISA Immunoassay kit for **(i)** CXCL10 and **(j)** IL‐6 in the supernatant of Calu‐3 and HMVEC‐L co‐cultured cells with mock or SARS‐CoV‐2 infection at 72 h after infection. *n* = 4 independent experiments. Data are presented as mean ± s.e.m. with individual data points indicated and colour coded per independent experimental replicate. Statistical significance was determined using the Mann–Whitney *U*‐test between mock and + SARS‐CoV‐2 **(b, d, f, g, h, i, j)**. *P < 0.05, **P < 0.01, ****P < 0.0001.

To create a more physiologically relevant *in vitro* model of the pulmonary endothelium, we adapted our previously described co‐culture model of the lung epithelial‐endothelial barrier,^52^ where epithelial (Calu‐3) cells are seeded on top of a transwell membrane, while endothelial cells (HMVEC‐L) are seeded on the basolateral side (Figure 6c). To mirror a respiratory infection, SARS‐CoV‐2 was added to the upper compartment and both epithelial and endothelial cells were analysed at 72 h post‐infection. In contrast to our observations in endothelial cell monocultures, no SARS‐CoV‐2 viral nucleocapsid protein or viral dsRNA was detected in HMVEC‐L, whereas nucleocapsid protein and viral dsRNA (indicating actively replicating virus) were clearly detected in infected Calu‐3 cells (Figure 6a, b, e and f). These results were confirmed by the presence of higher levels of viral RNA as measured by qPCR in the Calu‐3 compared to the HMVEC‐L (Figure 6g) and higher titres of infectious virions in the supernatants of the upper (Calu‐3) compartment of the transwell (Figure 6h). Despite the lack of endothelial infection, HMVEC‐L cells expressed ICAM‐1 when virus was added to the upper (Calu‐3) compartment (Figure 6a and d). HMVEC‐L also responded to infected Calu‐3 cells by increasing CXCL10 secretion (detected in lower compartment of the co‐culture, Figure 6i). Induction of ICAM‐1 and CXCL10 suggests that the HMVEC‐L respond to adjacent epithelial infection. Both Calu‐3 and HMVEC‐L appeared to secrete IL‐6 in response to SARS‐CoV‐2 infection, as IL‐6 levels were increased in both compartments (significantly in the upper, trending in the lower, Figure 6j). In summary, in a co‐culture model of the pulmonary epithelium, endothelial cells are not directly infected with the virus, but respond to adjacent epithelial infection by upregulating ICAM‐1 expression and releasing pro‐inflammatory cytokines. This suggests that endothelial inflammation and dysfunction during *in vivo* SARS‐CoV‐2 infection is most likely to occur as an indirect response to infection of the adjacent epithelial layer.

## DISCUSSION

SARS‐CoV‐2 can induce vascular dysfunction and thrombotic events in patients with severe COVID‐19. However, the cellular and molecular mechanisms by which SARS‐CoV‐2 infection triggers such pathologies have so far remained elusive. Here, we have shown that endothelial cells do not support productive replication of SARS‐CoV‐2, but that they induce pro‐inflammatory cytokines and adhesion molecules upon either direct or indirect exposure to SARS‐CoV‐2 virions. This supports the hypothesis that the endothelial dysfunction observed in COVID‐19 patients is likely to be largely mediated by inflammatory signalling pathways.

As COVID‐19 case numbers rose, reports of COVID‐19 coagulopathy and vascular dysfunction also began to accumulate. Whether SARS‐CoV‐2 directly infects the vasculature to drive this vascular pathology has thus remained a topic of intense debate. Multiple studies show viral particles surrounding the vasculature,^3^, ^29^, ^30^ yet it is unclear whether this infection is truly specific to the endothelium or occurs within the perivascular compartment. Our *in vivo* analyses of lung tissue samples from deceased COVID‐19 patients indicate that the endothelium remains uninfected. Nevertheless, we showed that *in vitro,* endothelial cells are theoretically susceptible to SARS‐CoV‐2 infection, as they express ACE2, TMPRSS2 and NRP1, albeit at significantly lower levels than epithelial cells. This is in line with other studies that showed expression of SARS‐CoV‐2 receptors by the endothelium in human tissue or in cultured cells.^15^, ^18^, ^24^, ^32^ However, we did not detect endothelial cell infection following exposure to 6 × 10^4^ PFUs of SARS‐CoV‐2, whereas epithelial cells were readily infected at the same viral dose. These results support those of Wang *et al*.,^32^ who showed that epithelial cells are more susceptible to infection than endothelial cells. We note that endogenous ACE2 in endothelial cells appeared to be glycosylated (˜120 kDa), while the ACE2 on airway epithelial cells (primary nasal epithelial cells and Calu‐3) appeared to be deglycosylated (˜98 kDa). It is tempting to speculate that the glycosylation status of ACE2 may determine whether cells can be infected with SARS‐CoV‐2, and that the glycosylated ACE2 on endothelial cells precludes efficient viral binding and uptake. Nevertheless, when exposed to high viral titres, endothelial cells became positive for viral nucleocapsid protein, suggesting effective viral entry. However, no infectious virions were detected in the endothelial cell supernatant, indicating that this infection was abortive. It is important to note that our results have been interpreted from a model of the lower respiratory tract, and the precise viral titres in this compartment of the respiratory tract are unclear.

Our ACE2 overexpression experiments suggest that a lack of functional ACE2 protein, in the form of deglycosylated ACE2, on HUVEC and HMVEC‐L is the primary factor limiting productive SARS‐CoV‐2 infection and replication in endothelial monocultures. Nevertheless, when monocultures of endothelial cells were exposed to high viral titles, we observe virus entering these cells even without ACE2 overexpression; however, this appears to be abortive and induces endothelial cell apoptosis. It is interesting to speculate why SARS‐CoV‐2 infection is not productive in this context. It may be that the ACE2 independent cell entry route is a cellular ‘dead end’, potentially with virus becoming trapped in subcellular compartments to prevent replication. Even with overexpressed ACE2 in endothelial cells, there may still be cell dependent differences in viral entry. For example, in epithelial cells, there may be sufficient surface TMPRSS2 levels available to cleave the spike protein at the S2’ site and mediate viral‐host cell membrane fusion. This may in turn allow for efficient release of viral RNA into the cytoplasm and thus viral replication. In contrast, in cells with lower TMPRSS2 expression (such as endothelial cells), SARS‐CoV‐2 may enter the cell via endocytosis, with S2’ cleavage being mediated by endosomal cathepsins.^13^ Endosomal entry may not only be inefficient, but it may also activate the cellular anti‐viral response and thereby limit productive viral replication.^53^, ^54^ Nevertheless, our overexpression data suggest that if endothelial cells express deglycosylated ACE2 at a sufficient level, then they can support productive SARS‐CoV‐2 replication.

In contrast to endothelial cells grown in a monoculture, viral proteins could not be detected in endothelial cells grown in a co‐culture with SARS‐CoV‐2‐positive epithelial cells, despite being exposed to the same dose of virus. These data may reflect the fact that SARS‐CoV‐2 typically infects and buds from epithelial cells in an apical manner, resulting in limited exposure of the endothelium to infectious virions. Extrapolating these data to the *in vivo* situation would suggest that a basolateral (abortive) infection of the endothelium by SARS‐CoV‐2 is only likely to occur when the epithelial barrier of the lung is severely damaged, exposing the endothelium to incoming virions.

While endothelial cells (either in a monoculture or in a co‐culture) did not produce infectious virions, they did respond to challenge with SARS‐CoV‐2. Analysis of infected endothelial monolayers revealed that cells positive for viral proteins were extruded in an apical fashion, without any apparent disruption to the monolayer, as assessed by F‐actin (Phalloidin) immunostaining. This is in contrast to work from Buzhdygan and colleagues, who demonstrated that purified SARS‐CoV‐2 spike protein can induce the breakdown of the vascular barrier in brain microvascular endothelial cells.^55^ Discrepancies between these results may be because of different sources of primary endothelial cells (lung versus brain), or the differences in response of the endothelium to a purified viral protein versus infectious virus.^1^, ^3^, ^25^, ^30^, ^56^, ^57^, ^58^ However, if infected cells are promptly removed from exposed vessels as our data suggest, then this may explain why detection of viral particles in the endothelium of patients has varied between studies.^4^, ^59^

In the absence of direct endothelial infection, hyperinflammation is likely to contribute to endothelial dysfunction observed in COVID‐19 patients. SARS‐CoV‐2 triggered an upregulation of endothelial ICAM‐1 expression when HMVEC‐Ls were directly exposed to virus, or co‐cultured with infected epithelial cells. ICAM‐1 enables immune cells to effectively extravasate into tissues, so its upregulation is expected to facilitate the observed influx of inflammatory monocytes, neutrophils and other immune cells into the lungs of severe COVID‐19 patients.^60^ This suggests that locally produced pro‐inflammatory cytokines stimulate the endothelium to induce adhesion marker expression. The precise suite of cytokines or epithelial factors responsible for inducing this endothelial cell response remains to be determined. Infected epithelial cells are likely to produce key cytokines such as IL‐6, Type I and III IFNs and CXCL10, but dying epithelial cells may also release cellular alarmins or viral components (e.g. RNA, viral proteins) that contribute to inflammatory responses. We observed that inflamed endothelial cells remain refractory to productive SARS‐CoV‐2 infection (Figure 5h–j), suggesting the vasculature is no more likely to become infected in patients with severe disease. Our previous studies with influenza virus showed that endothelial cells can themselves be a source of these pro‐inflammatory cytokines,^61^ and indeed, we also observed SARS‐CoV‐2‐induced release of IL‐6 and CXCL10 in co‐cultures. Precisely how SARS‐CoV‐2 is sensed by the endothelium in either the monoculture or co‐culture system remains to be determined. Given that SARS‐CoV‐2 does not replicate in endothelial cells, we speculate that endothelial cells are unlikely to sense the virus through cytosolic RNA receptors such as RIG‐I or MDA5, as these immune sensors would detect viral replication intermediates. A more likely scenario is that the endothelial cells respond to danger signals from neighbouring infected epithelial or endothelial cells. These data add to a growing body of literature that indicates endothelial cell directed inflammation plays a key role in the pathogenesis of SARS‐CoV‐2.^62^ It is tempting to speculate that, similar to influenza virus, the SARS‐CoV‐2‐induced pro‐inflammatory state may induce endothelial cells to express tissue factor, which in turn induces a pro‐coagulant state, microvascular leakage and pulmonary haemorrhage,^63^ all of which have been described in patients with severe COVID‐19. Furthermore, regardless of direct or indirect infection, elevated IL‐6 correlates with increased fibrinogen^64^ and although still controversial, elevated fibrinogen levels have been detected in critically ill patients.^2^

The present study was subject to several limitations. In addition to endothelial dysfunction,^3^ the lungs of deceased COVID‐19 patients show evidence of coagulation, thrombosis and induction of angiogenesis. Our study has not addressed the effect of SARS‐CoV‐2 exposure on the angiogenic capacity of endothelial cells. This effect appears to be specific to COVID‐19 patients, as lungs from deceased influenza patients do not display increased angiogenic features. This angiogenic response is thought to be predominantly mediated through intussusceptive angiogenesis, where a new blood vessel is formed by the splitting of an existing vessel. Whether this is because of relative hypoxia in the lungs remains unclear, although elevated angiogenic growth factors such as VEGF‐A and VEGF‐C are associated with COVID‐19.^3^, ^49^ Given the intimate association between inflammation, endothelial dysfunction and angiogenesis,^50^, ^65^ these are critical aspects of COVID‐19 pathology that remain to be addressed in subsequent studies. Similarly, because of the very low levels of ACE2 present on endothelial cells, it was not possible to determine whether this was more pronounced on the apical or basolateral surface of the endothelial cell.

An additional limitation of the present study is that, because of the reductionist nature of the *in vitro* systems used herein, we were unable to address the contribution of immune cells to endothelial cell dysfunction during SARS‐CoV‐2 infection. Multiple studies attribute the sustained inflammatory response directly to immune cells, wherein macrophage activation, monocyte NLRP3 inflammasome signalling, complement activation and extrusion of neutrophil extracellular traps are implicated.^51^, ^66^, ^67^, ^68^, ^69^ Accordingly, our data may best reflect the initial stages of infection when only a limited number of tissue‐resident leukocytes may be present. Future studies will necessitate the addition of leukocytes to co‐culture systems, to determine whether their presence results in further induction of inflammation and markers of severe disease. Finally, while Calu‐3 cells are a well‐accepted model of respiratory epithelial cells in the context of SARS‐CoV‐2,^43^ future studies would ideally utilise primary airway epithelial cells in co‐culture with primary endothelial cells to more accurately model the lung environment.

In summary, we have conclusively shown that *in vitro,* endothelial cells are not productively infected by SARS‐CoV‐2 but that they mount an inflammatory response after direct or indirect exposure to the virus, characterised by increased cytokine secretion and expression of adhesion molecules. Our results thus suggest a key role for the endothelium in the pathogenesis of COVID‐19, and that targeting the inflammatory response may present the best opportunity to prevent endothelial dysfunction.

## METHODS

### *In vivo* samples

Lung tissue from 10 SARS‐CoV‐2 infected patients was sectioned and Haematoxylin and Eosin (H&E) staining performed. RNAscope probes (ACDbio, US) targeting SARS‐CoV‐2 spike mRNA (#848561‐C3) were used as per the manufacturer’s instructions for automation on Leica Bond RX. DNA was visualised with Syto13, and SARS‐CoV‐2 spike probe with opal 690 (1:1500). Fluorescent images were acquired with Nanostring Mars prototype DSP at 20×. Autopsy and biopsy materials were obtained from the Pontificia Universidade Catolica do Parana PUCPR the National Commission for Research Ethics (CONEP) under ethics approval numbers 2020001792/30188020.7.1001.0020 and 2020001934/30822820.8.000.0020. The study was also approved under The University of Queensland HREC ratification.

### Cell culture

Human umbilical vein endothelial cells (HUVECs) and human microvascular endothelial cells from lungs (HMVEC‐L) purchased from Lonza (CC‐2935 and CC‐2527 respectively) were cultured until passage 8 in EGM‐Plus or EGM‐2MV medium, supplemented with singlequots (Lonza CC‐5035, CC‐3102). Calu‐3 cells purchased from ATCC (HTB‐55) were maintained in MEM (Invitrogen), containing 10% (v/v) heat‐inactivated foetal bovine serum (Cytiva), 100 U mL^−1^ penicillin and streptomycin (Life Technologies Australia), and grown in EGM‐2MV for endothelial co‐culture experiments. Baby hamster kidney cells (BHK‐21) were a kind gift from Professor RG Parton. Cell lysates of human nasal epithelial cells (HNEp) were a kind gift from Professor Peter Sly. Human Embryonic Kidney (HEK)‐293T cells were maintained in DMEM with L‐glutamine and sodium pyruvate (Invitrogen), containing 10% (v/v) heat‐inactivated foetal bovine serum (GE healthcare Australia), 100 U per mL penicillin and streptomycin (Life Technologies Australia). All cells were cultured at 37°C and 5% CO_2_.

Monocultures of HUVEC, HMVEC‐L or Calu‐3 cells were performed on 24‐well cell culture inserts (Corning 6.5 mm Transwell, 0.4 µm polycarbonate membrane 3413) coated with 5 µg mL^−1^ fibronectin (FN) (Sigma). Approximately 25,000 HUVEC, 25,000 HMVEC‐L and 50,000 Calu‐3 cells were seeded per insert on either the apical or basolateral side as indicated and grown in EGM‐Plus (Lonza), EGM‐2MV (Lonza) or MEM (Invitrogen) respectively. Endothelial cells were treated with 100 ng mL^−1^ TNFα (Life Technologies Australia) or 10 ng mL^−1^ IFNß (Lonza) for the times indicated.

Calu‐3 and HMVEC‐L were co‐cultured on 24‐well cell culture inserts (Corning 6.5 mm Transwell, 0.4 µm polycarbonate membrane Cat#3413) coated with 5 µg mL^−1^ FN (Sigma) in EGM‐2MV (Lonza). Approximately 25,000 HMVEC‐L were seeded on an inverted transwell membrane for 1 h to attach. Transwell membranes were turned over to normal position, and approximately 50,000 Calu‐3 cells were seeded in the top compartment of the membrane. Cells were grown for 48 h prior to infection.

### Viral infection

SARS‐CoV‐2 isolate hCoV‐19/Australia/QLD02/2020 was provided by Queensland Health Forensic & Scientific Services, Queensland Department of Health. Virus was grown on Vero cells and titred.^70^ Sanger sequencing was used to confirm that no mutations occurred in the spike gene relative to the original clinical isolate. Cells were infected with 6 × 10^4^ plaque‐forming units (PFUs) for 1 h at 37°C 5% CO_2_. Virus was added in a volume of 250 mL. The viral inoculum was then removed, and the medium was replaced with DMEM (Invitrogen) or MEM (Invitrogen) containing 2% FBS. Alternatively, 2 × 10^6^ PFUs were added to the cell culture and cells were incubated at 37°C 5% CO_2_. All studies with SARS‐CoV‐2 were performed under physical containment 3 (PC3) conditions and were approved by The University of Queensland Biosafety Committee (IBC/374B/SCMB/2020).

### Lentiviral transduction

A lentiviral construct containing human ACE2 (Addgene 155295) was packaged into lentivirus in HEK‐293T cells by means of third generation lentiviral packaging plasmids.^71^ Lentivirus containing supernatant was harvested on day 2 and 3 after transfection. Lentivirus was concentrated by Lenti‐X concentrator (Clontech, 631232). HEK cells were transfected with the expression vectors according to the manufacturer’s protocol with PEI 2500 (BioScientific) and transduced target HMVEC‐L cells were selected with puromycin (1 mg mL^−1^) after 24 h and used for assays after 72 h.

### Antibodies

The following antibodies were used: mouse anti‐ACE2 (Santa Cruz, sc‐390851, IF1:200), goat anti‐ACE2 (R&D systems, AF933, WB 1:1000), mouse anti‐dsRNA (Millipore, MABE1134, IF1:100), rabbit anti‐SARS‐CoV‐2 nucleocapsid protein/NP (Sino Biological, 40143‐R040, WB1:1000 IF1:200), rabbit anti‐TMPRSS2 (Abcam, ab92323, WB1:1000), rabbit anti‐cleaved caspase 3 (Cell Signaling, 9661 IF1:30 after pre‐labelling), rabbit anti‐GAPDH (Cell Signaling, 2118, WB1:2000), mouse anti‐ICAM‐1 (R&D systems, BBA3, IF1:200), mouse anti‐ICAM‐1 (Santa Cruz Biotechnology, sc‐8439, WB1:1000), Phalloidin‐Alexa555 (Cytoskeleton, PHDH1‐A, IF1:500), Phalloidin‐Alexa670 (Cytoskeleton, PHDN1‐A, IF1:200). Pre‐labelling of rabbit anti‐cleaved caspase 3 with Alexa Fluor 555 was performed using Zenon Rabbit IgG labelling kit (ThermoFisher, Z‐25305) according to manufacturer protocol.

### Immunofluorescence staining

Immunofluorescence staining was in general performed on cells cultured on 12‐mm glass coverslips or on 24‐well cell culture inserts (Corning 6.5 mm Transwell, 0.4 µm polycarbonate membrane 3413) coated with 5 µg mL^−1^ FN (Sigma), washed with PBS^+/+^ (PBS supplemented with 1 mM CaCl_2_ and 0.5 mM MgCl_2_), fixed for 10 min in 4% PFA (Sigma), blocked and permeabilised for 30 min with 3% BSA, 0.3% Triton X‐100 (Sigma). Primary antibodies were incubated in 1.5% BSA for 1 h at RT, and secondary antibodies linked to Alexa fluorophores (all Invitrogen) were incubated in 1.5% BSA for 1 h at RT and incubated with 4% PFA (Sigma) for another 24 h in case of SARS‐CoV‐2‐infected cells, after each step samples were washed 3× with PBS^+/+^ and mounted with ProlongGold + DAPI solution (Cell Signaling Technologies, CST 8961S). Z‐stack image acquisition was performed on a confocal laser scanning microscope (Zeiss LSM880) using a 40× NA 1.3 or 63× NA 1.4 oil immersion objective.

### 3D Microfluidic vessel culture

Microfluidic devices were generated as previously described^48^ using 2.5 mg mL^−1^ Collagen (R&D Systems) for the extracellular matrix. Cells were seeded 48 h prior to virus infection and were exposed to oscillatory shear stress by virtue of being kept on a rocker at 37°C and 5% CO_2_. The tubes were fixed sequentially in 1% formaldehyde, containing 0.05% Triton for 90 s, followed by 4% formaldehyde for 30 min at 37°C. Tubes were permeabilised with 0.5% Triton for 10 min at 37°C and blocked for 4 h in 10% goat serum in PBS at 4°C prior to staining. Z‐stack image acquisition was performed on a confocal laser scanning microscope (Zeiss Axiovert 200 inverted microscope with LSM 710 Meta Confocal Scanner) using 40× NA 1.1 water immersion objective.

### Western blotting

For total cell lysates, cells were washed once with PBS and lysed with RIPA buffer (50 mM Tris, 150 mM NaCl, 1 mM EDTA, 1% Triton X‐100, 0.1% SDS, 1% sodium deoxycholate, protease inhibitor, pH 8.0). A Pierce BCA protein assay kit (Thermo Scientific) was used to equalise protein amounts and SDS‐sample buffer containing 100 mM DTT (Astral Scientific) was added, and samples were boiled at 95°C for 10 min to denature proteins. Proteins were separated on 4‐15% mini protean TGX precast gels (Bio‐Rad) in running buffer (200 mM Glycine, 25 mM Tris, 0.1% SDS (pH8.6)), transferred to nitrocellulose membrane (Bio‐Rad 1620112) in blot buffer (48 nM Tris, 39 nM Glycine, 0.04% SDS, 20% MeOH) and subsequently blocked with 5% (w/v) BSA in Tris‐buffered saline with Tween‐20 (TBST) for 30 min. The immunoblots were analysed using primary antibodies incubated overnight at 4°C and secondary antibodies linked to horseradish peroxidase (HRP) (Invitrogen), and after each step, immunoblots were washed 4× with TBST. HRP signals were visualised by enhanced chemiluminescence (ECL) (Bio‐Rad) and imaged with a Chemidoc (Bio‐Rad).

### RNA extraction and cDNA synthesis

RNA was extracted using the Qiagen RNeasy Minikit (Qiagen) or NucleoZOL (BIOKÉ, The Netherlands) according to the manufacturer’s guidelines. cDNA was synthesised using random hexamers and the high‐capacity cDNA reverse transcription kit (Life Technologies) according to the manufacturer’s guidelines.

### Quantitative PCR (qPCR)

qPCR was performed using SYBR Green reagent (Applied Biosystems). All primer sequences are listed in Supplementary table 2. qPCR conditions were applied according to the manufacturer’s instructions using QuantStudio 6 Flex Real‐Time PCR System (ThermoFisher). HPRT or Glyceraldehyde 3‐phosphate dehydrogenase (GAPDH) was used as a housekeeping gene and relative gene expression was determined using the comparative cycle threshold (ΔΔCt) method (Livak, 2001).^72^

### Viral titres

Viral titres in cell culture supernatants were determined by a plaque assay on Vero cells, according to previously described protocols.^70^

### Cytokine titres

Cytokine titres were determined using an AlphaLISA Immunoassay kit (Perkin Elmer) according to the manufacturer’s instructions. The sum of cytokines detected in the upper and lower compartments of the transwell is shown for each of the monocultured samples, while cytokine levels were kept separated for the upper and lower compartment for the co‐cultured samples.

### Image analysis

Analysis of immunofluorescent images was performed using ImageJ version 2.0.0‐rc‐69/1.52n. A mask of the total cell area was created using default intensity threshold on Phalloidin staining. Within the mask, Raw Integrated Density of either ACE2, NP or ICAM‐1 was measured, and value was corrected for total mask area resulting in RawIntDen/Area. For dsRNA, MaxEntropy threshold was performed on dsRNA staining to remove background speckles before measuring Raw Integrated Density within the mask. Analysis of Western blot images was performed using Gel Analyzer Tool of ImageJ version 2.0.0‐rc‐69/1.52n. The background signal was subtracted, and values were normalised to corresponding GAPDH loading control. Imaris (Bitplane) version 8 was used to create the XZ projection in Figure 3b. Extrusion of cells was quantified in Figure 3c using orthogonal views in ImageJ version 2.0.0‐rc‐69/1.52n.

### Statistical analysis

All statistical analysis was performed using GraphPad Prism version 9.0. Data are presented as mean ± s.e.m. with individual data points indicated and colour coded per independent experimental replicate. Statistical significance was determined using the Kruskal–Wallis test for multiple comparisons or the Mann–Whitney *U*‐test between mock and SARS‐CoV‐2‐treated conditions. *P < 0.05, **P < 0.01, ***P < 0.001 and ****P < 0.0001.

## Conflict of interest

KS is a co‐inventor on patent applications for NLRP3 inhibitors, which have been licensed to Inflazome Ltd, a company headquartered in Dublin, Ireland. Inflazome is developing drugs that target the NLRP3 inflammasome to address unmet clinical needs in inflammatory disease. KS served on the Scientific Advisory Board of Inflazome in 2016–2017, and serves as a consultant to Quench Bio, USA, and Novartis, Switzerland.

## Author contributions

**Lilian Schimmel:** Conceptualization; formal analysis; investigation; methodology; validation; visualization; writing‐original draft; writing‐review & editing. **Keng Yih Chew:** Conceptualization; formal analysis; investigation; methodology; validation; writing‐review & editing. **Claudia J Stocks:** Formal analysis; investigation; validation. **Teodor Yordanov:** Investigation; methodology; validation. **Patricia Essebier:** Investigation; validation. **Arutha Kulasinghe:** Formal analysis; investigation; methodology; resources. **James Monkman:** Investigation; resources. **Anna Flavia Ribeiro dos Santos Miggiolaro:** Resources. **Caroline Cooper:** Resources. **Lucia de Noronha:** Resources. **Kate Schroder:** Conceptualization; funding acquisition; supervision; writing‐original draft; writing‐review & editing. **Anne K Lagendijk:** Conceptualization; funding acquisition; methodology; supervision; writing‐original draft; writing‐review & editing. **Larisa I Labzin:** Conceptualization; formal analysis; funding acquisition; investigation; methodology; supervision; validation; visualization; writing‐original draft; writing‐review & editing. **Kirsty R Short:** Conceptualization; funding acquisition; methodology; project administration; supervision; writing‐original draft; writing‐review & editing. **Emma J Gordon:** Conceptualization; funding acquisition; methodology; project administration; supervision; writing‐original draft; writing‐review & editing.

## Supporting information

Supplementary tables 1‐2
Supplementary figures 1‐6

## Data Availability

All raw data are available upon request.
