# Supplementary material for: Endothelial cells are not productively infected by SARS‐CoV‐2
Source: Clin Transl Immunology. 2021 Oct 24;10(10):e1350. doi: 10.1002/cti2.1350 (PMC8542944; doi:10.1002/cti2.1350)
Supplement: Supplementary file 1 — Supplementary tables 1‐2 Supplementary figures 1‐6 [file CTI2-10-e1350-s001.pdf]

Supplementary table 1: Patient information

Patient ID	Diagnosis	Sex	Age	Co-morbidities	Histology	Computed tomography chest at admission	Length of stay on mechanical ventilation	Admission to death
PT1*	COVID-19	F	87	Systemic Arterial Hypertension, Dyslipidemia, Hypothyroidism, Senile dementia	Acute phase DAD	Diffuse and bilateral “opacities with ground-glass attenuation”, thickening of the pulmonary septum, suggestive of viral pulmonary infection	8 days	8 days
PT2	COVID-19	M	53	Class II obesity	Acute phase DAD	Diffuse and bilateral “opacities with ground-glass attenuation”, suggestive of viral pulmonary infection	8 days	13 days
PT3*	COVID-19	F	85	Systemic Arterial Hypertension, Brain Stroke, Vascular Dementia	Acute phase DAD	Image of “opacities with ground-glass attenuation” upper left lobe, suggestive of viral pulmonary infection, and small volume pleural effusion	0 days	23 days
PT4	COVID-19	M	73	Type 2 Diabetes Mellitus, Chronic Kidney Disease Dialysis, Atrial Fibrillation, Coronary Disease, Heart Failure, Peripheral Obstructive Artery Disease	Reactive Type 2 pneumocyte hyplerplasia	Diffuse and bilateral “opacities with ground-glass attenuation”, suggestive of viral pulmonary infection	10 days	38 days
PT6	COVID-19	M	80	Systemic Arterial Hypertension, Coronary Disease, Heart Failure, Class III obesity	Reactive Type 2 pneumocyte hyplerplasia	Diffuse and bilateral “opacities with ground-glass attenuation”, suggestive of viral pulmonary infection	21 days	23 days

PT7	COVID-19	M	81	Systemic Arterial Hypertension, Chronic Kidney Disease Dialysis, Brain Stroke	Reactive Type 2 pneumocyte hylperplasia	Diffuse and bilateral “opacities with ground-glass attenuation”, suggestive of viral pulmonary infection	8 days	8 days
PT8	COVID-19	N/A	N/A	N/A	Organising pneumonia	N/A	N/A	N/A
PT9	COVID-19	M	86	Prostate cancer, Abdominal Aortic Aneurysm, Giant Cells Arteritis	Organising pneumonia	Peripheral, multifocal and bilateral “opacities with ground-glass attenuation”, thickening of the pulmonary septum, suggestive of viral pulmonary infection	3 days	N/A
PT10	COVID-19	M	46	Dyslipidemia	Organising pneumonia	Peripheral, multifocal and bilateral “opacities with ground-glass attenuation”, suggestive of viral pulmonary infection	5 days	N/A
PT11	COVID-19	F	93	Type 2 Diabetes Mellitus, Systemic Arterial Hypertension, Dyslipidemia, Senile dementia	Acute phase DAD	Diffuse and bilateral “opacities with ground-glass attenuation”, thickening of the pulmonary septum, suggestive of viral pulmonary infection	6 days	N/A

*representative images shown in Supplementary Figure 1b, c derived from this patient

Supplementary table 2: Primers used in the present study

Name	Sequence (5’-3’)
SARS-COV2 (MPRO) VIRUS FW	GAGACAGGTGGTTTCTCAATCG
SARS-COV2 (MPRO) VIRUS RV	ACGGCAATTCCAGTTTGAGC
GAPDH FW	CGAGATCCCTCCAAAATCAA
GAPDH RV	TTCACACCCATGACGAACAT
HPRT FW	TCAGGCAGTATAATCCAAAGATGGT
HPRT RV	AGTCTGGCTTATATCCAACACTTCG
ACE2 FW	TCACGATTGTTGGGACTCTGC
ACE2 RV	TCGCTTCATCTCCCACCACT
NEUROPILIN FW	TGGACCGACCACTCCCAACG
NEUROPILIN RV	AGAGCCCCAGCCAAATTCACAG
TMPRSS2 FW	GGAAGTTCATGGGCAGCAAG
TMPRSS2 RV	AGGCGAACACACCGATTCTC

Supplementary Figure 1

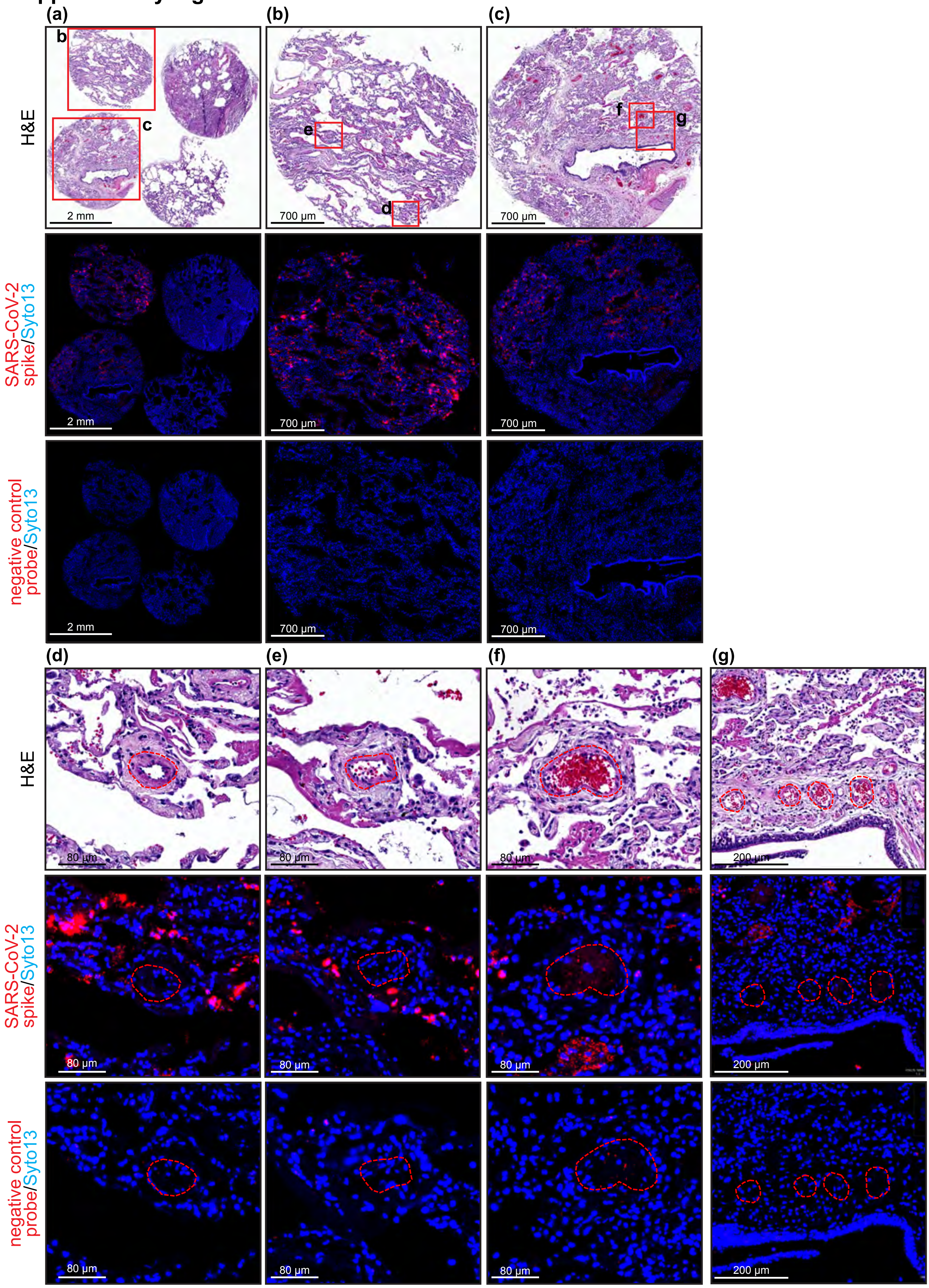

Supplementary figure 1. ***In vivo*** **sections of lung show endothelial cells are not infected.** **(a)** Lung tissue from 4 deceased COVID-19 patients was sectioned and stained for Hematoxylin and Eosin (H&E). Consecutive sections were stained using RNAscope probes targeting either SARS-CoV-2 spike mRNA or negative control probe and Syto13 for DNA. **(b-c)** Patients 1 and 2 are positive for SARS-CoV-2 spike mRNA. **(d-g)** Further enlargements of insets show vessels outlined with red dashed line are negative for SARS-CoV-2 spike mRNA in the endothelial layer of the vessels. Negative control probe shows non-specific background staining.

Supplementary figure 2

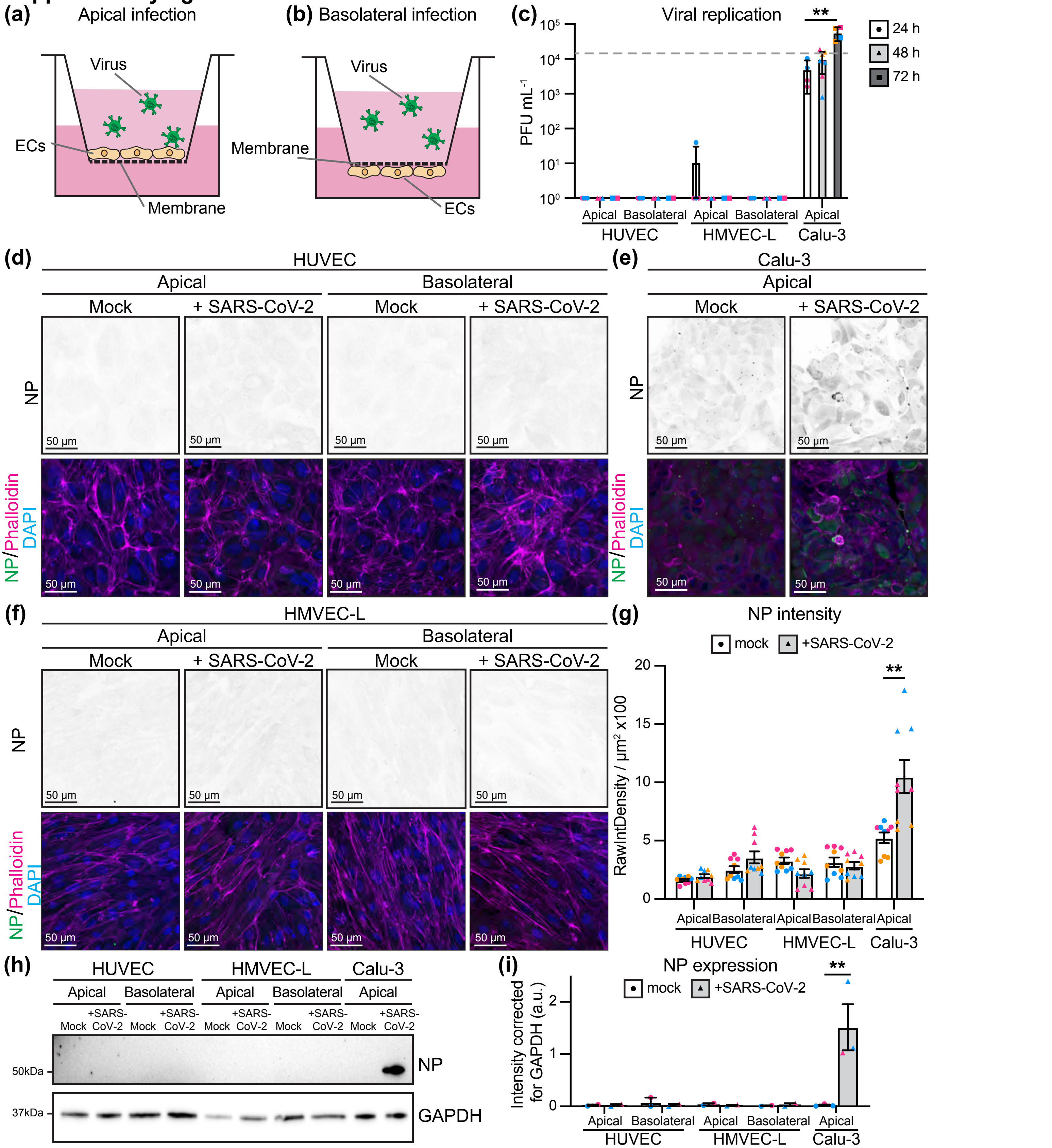

Supplementary figure 2. Endothelial cells are not productively infected with 6 x 10^4 PFU of SARS-CoV-2. Schematic of **(a)** apical and **(b)** basolateral infection of cells cultured on transwell membranes. **(c)** Viral replication shown as number of PFU ml^{-1} of supernatant from SARS-CoV-2 infected HUVEC, HMVEC-L and Calu-3 cells at 24h, 48h and 72h after infection. Grey dashed line indicates input level. n = 2 (HUVEC and HMVEC-L), n = 3 (Calu-3) independent experiments. Representative immunofluorescent images of **(d)** HUVEC, **(e)** Calu-3 or **(f)** HMVEC-L stained for NP (shown as single channel in top panel) (green), phalloidin (magenta) and DAPI (blue) with mock or SARS-CoV-2 infection from either apical or basolateral side of the cells at 48h after infection. Scalebar 50 μm. **(g)** Quantification of NP staining intensity in HUVEC, HMVEC-L and Calu-3. n = 9 images from 3 independent experiments. **(h)** Western blot analysis showing NP protein levels in HUVEC, HMVEC-L and Calu-3 cells after 48h of infection. **(i)** Quantification of protein levels for NP in HUVEC, HMVEC-L and Calu-3. n = 2 independent experiments. Data are presented as mean ± s.e.m. with individual data points indicated and colour coded per independent experimental replicate. Statistical significance was determined using Kruskal Wallis test between 24h and other time points (c) or Mann-Whitney-*U* test between mock and +SARS-CoV-2 (g,i). $*P < 0.05$, $**P < 0.01$.

Supplementary figure 3

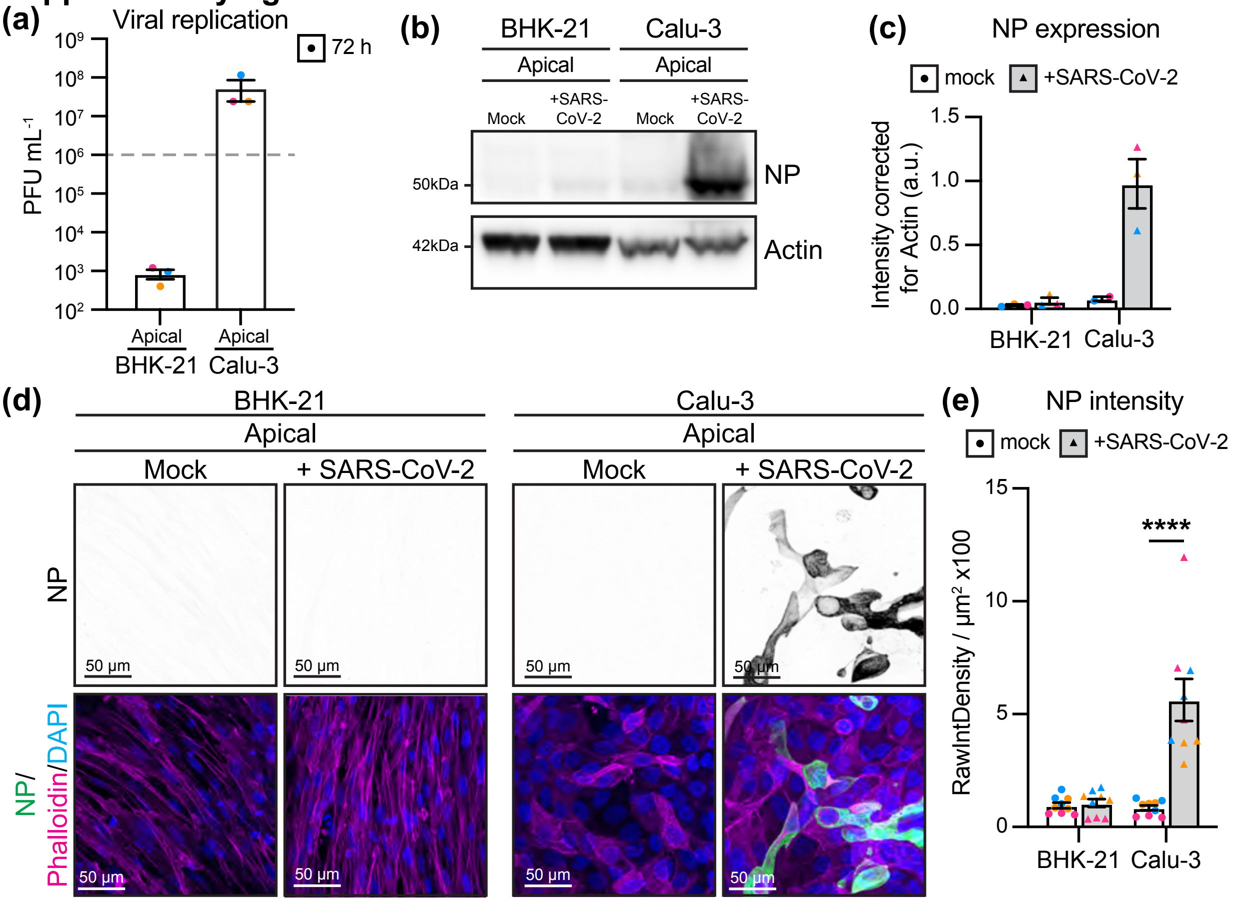

Supplementary figure 3. Positive and negative controls for infection with 2 x 10^6 PFU of SARS-CoV-2. (a) Viral replication shown as number of PFU per ml of supernatant from SARS-CoV-2 infected BHK-2 and Calu-3 cells at 72h after infection. Grey dashed line indicates input level. n = 3 independent experiments. **(b)** Western blot analysis showing NP protein levels in BHK-21 and Calu-3 cells after 72h of apical infection with mock or SARS-CoV-2. **(c)** Quantification of protein levels for NP in BHK-21 and Calu-3. n = 3 independent experiments. **(d)** Representative immunofluorescent images of BHK-21 and Calu-3 cells stained for NP (shown as single channel in top panel) (green), phalloidin (magenta) and DAPI (blue) with mock or SARS-CoV-2 infection from apical side of the cells at 72h after infection. Scalebar 50 μm. **(e)** Quantification of NP staining intensity in BHK-21 and Calu-3. n = 9 images from 3 independent experiments. Data are presented as mean ± s.e.m. with individual data points indicated and colour coded per independent experimental replicate. Statistical significance was determined using Mann-Whitney-*U* test between mock and +SARS-CoV-2 (c, e). $****P < 0.0001$.

Supplementary figure 4

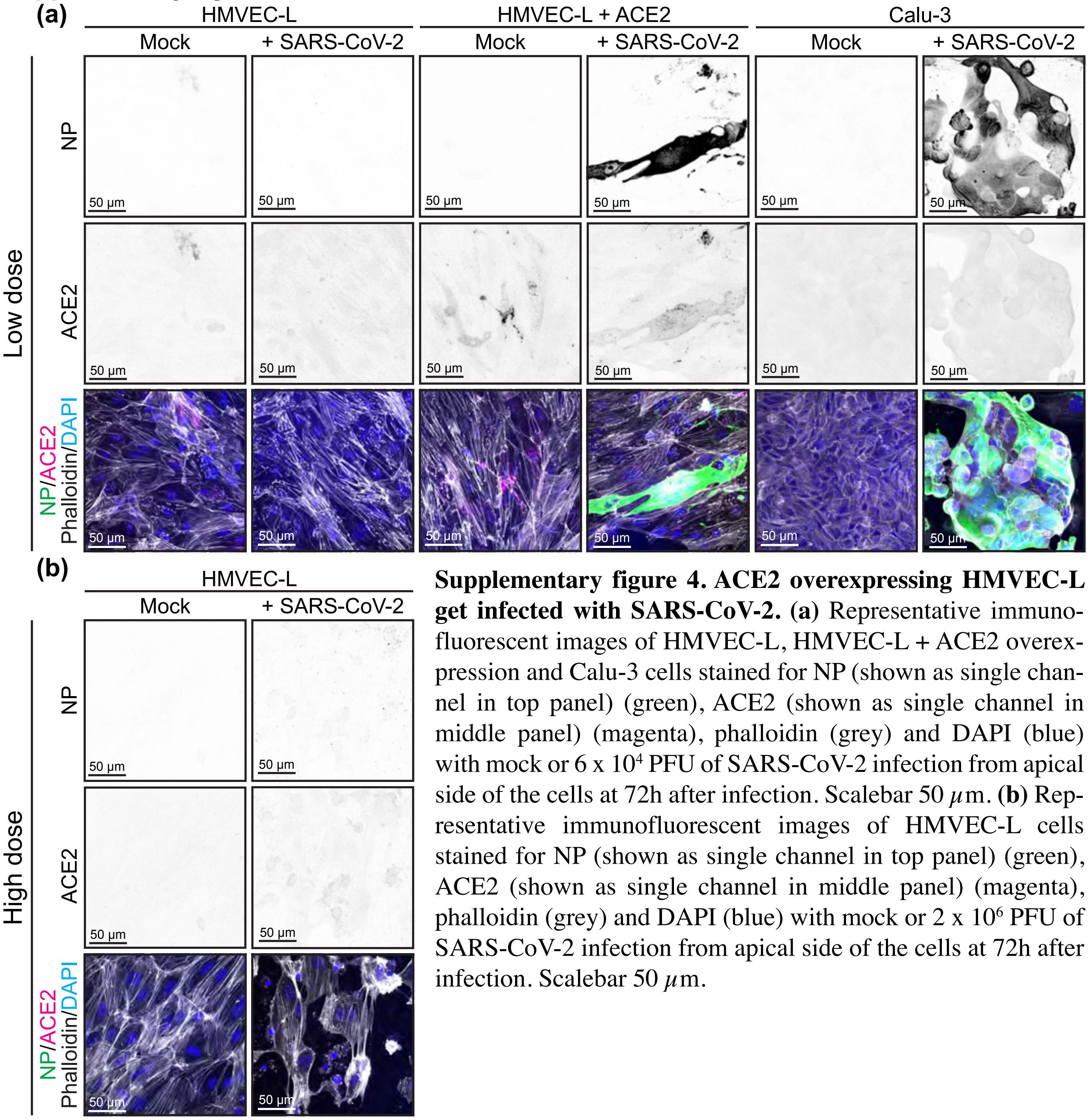

Supplementary figure 4. ACE2 overexpressing HMVEC-L get infected with SARS-CoV-2. (a) Representative immunofluorescent images of HMVEC-L, HMVEC-L + ACE2 overexpression and Calu-3 cells stained for NP (shown as single channel in top panel) (green), ACE2 (shown as single channel in middle panel) (magenta), phalloidin (grey) and DAPI (blue) with mock or 6 x 10^4 PFU of SARS-CoV-2 infection from apical side of the cells at 72h after infection. Scalebar 50 μm. **(b)** Representative immunofluorescent images of HMVEC-L cells stained for NP (shown as single channel in top panel) (green), ACE2 (shown as single channel in middle panel) (magenta), phalloidin (grey) and DAPI (blue) with mock or 2 x 10^6 PFU of SARS-CoV-2 infection from apical side of the cells at 72h after infection. Scalebar 50 μm.

Supplementary figure 5

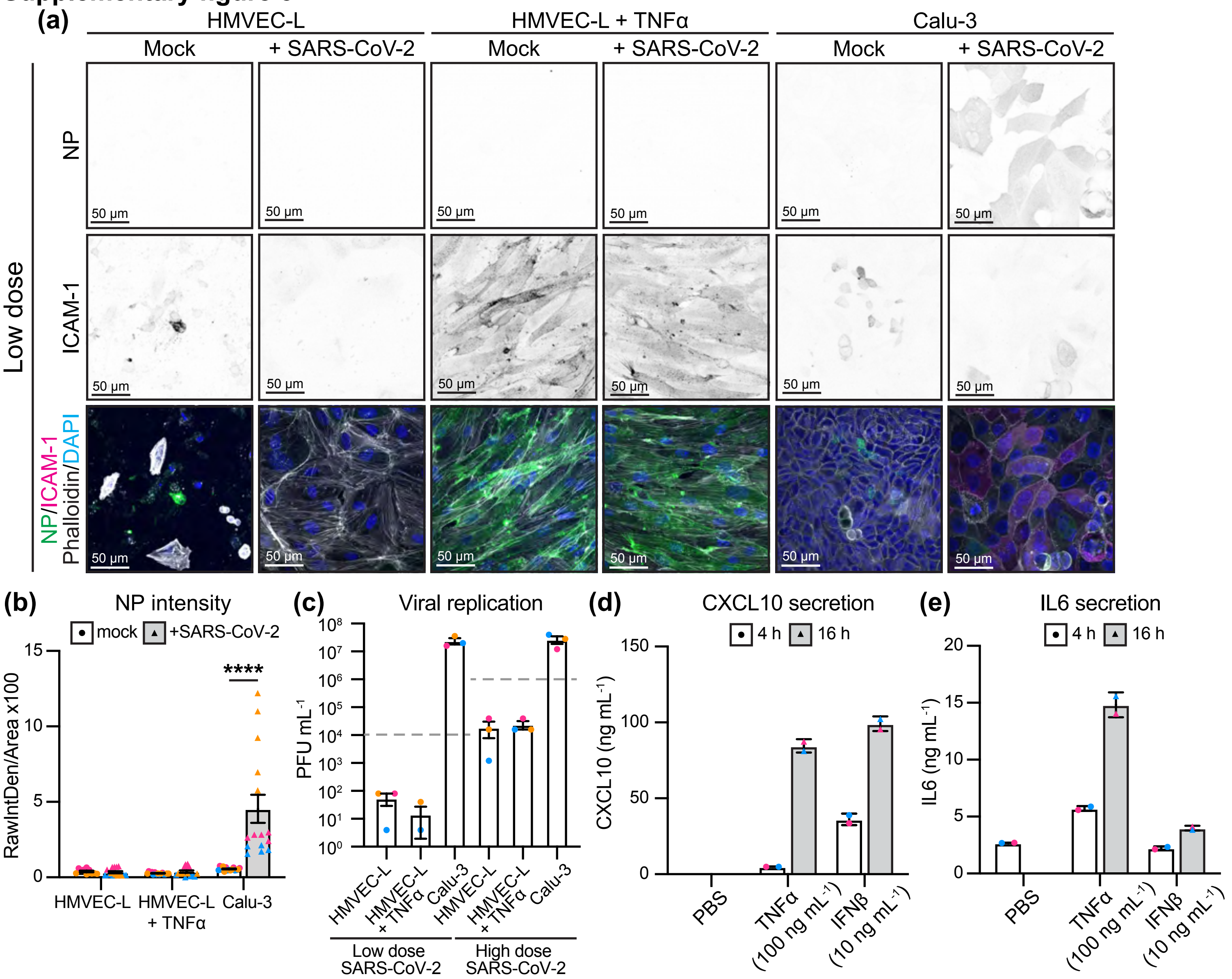

Supplementary figure 5. Inflamed HMVEC-L do not get infected with SARS-CoV-2 at low dose (6 x 10^4). (a) Representative immunofluorescent images of HMVEC-L, HMVEC-L + TNFα pre-treatment and Calu-3 cells stained for NP (shown as single channel in top panel) (magenta), ICAM-1 (shown as single channel in middle panel) (green), phalloidin (grey) and DAPI (blue) with mock or 6 x 10^4 PFU of SARS-CoV-2 infection from apical side of the cells at 72h after infection. Scalebar 50 μm. **(b)** Quantification of NP staining intensity in HMVEC-L, HMVEC-L + TNFα and Calu-3 with SARS-CoV-2 infection. n = 15 images from 3 independent experiments. **(c)** Viral replication shown as number of PFU mL^{-1} of supernatant from SARS-CoV-2 infected HMVEC-L, HMVEC-L + TNFα and Calu-3 cells at 72h after infection with either 6 x 10^4 PFU (low dose) or 2 x 10^6 PFU (high dose). Grey dashed line indicates input levels. n = 3 independent experiments. Maximum secretion levels of **(d)** CXCL10 and **(e)** IL6 in the supernatant of HMVEC-L with mock, TNFα or IFNß treatment from the apical side of the cells at 4h and 16h after treatment. n = 2 independent experiments.Data are presented as mean ± s.e.m. with individual data points indicated and colour coded per independent experimental replicate. Statistical significance was determined using Mann-Whitney-*U* test between mock and + SARS-CoV-2 (b). ****P < 0.0001.

Supplementary figure 6

Fig 1c

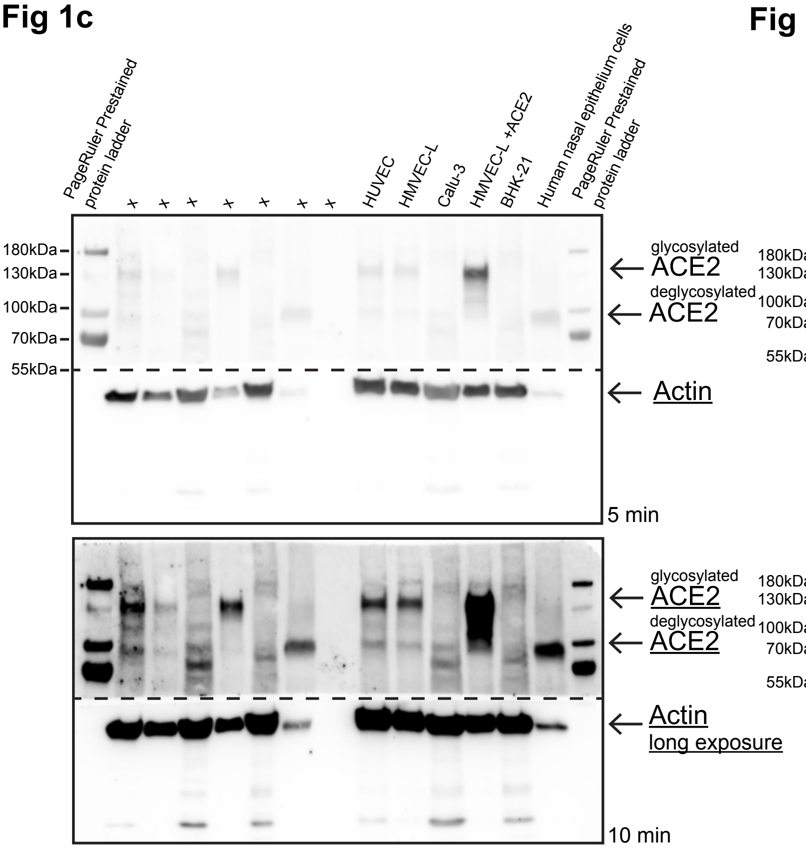

Fig 2d

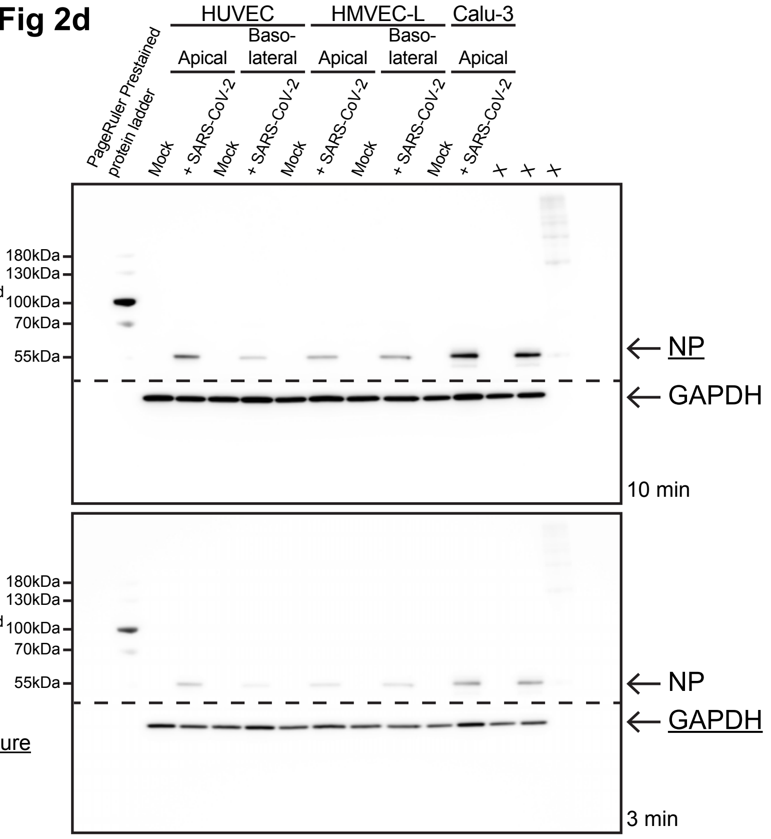

Fig 1d

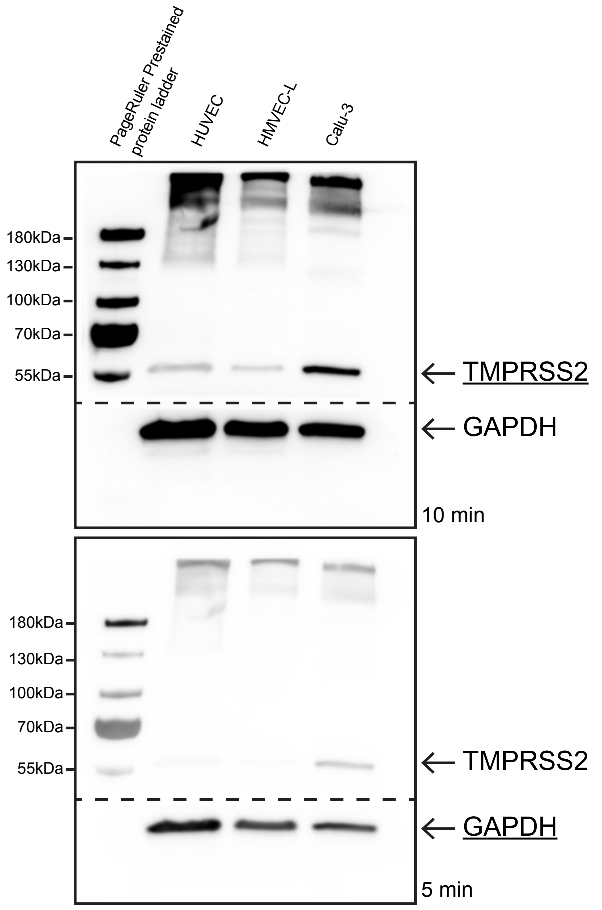

Fig 5d

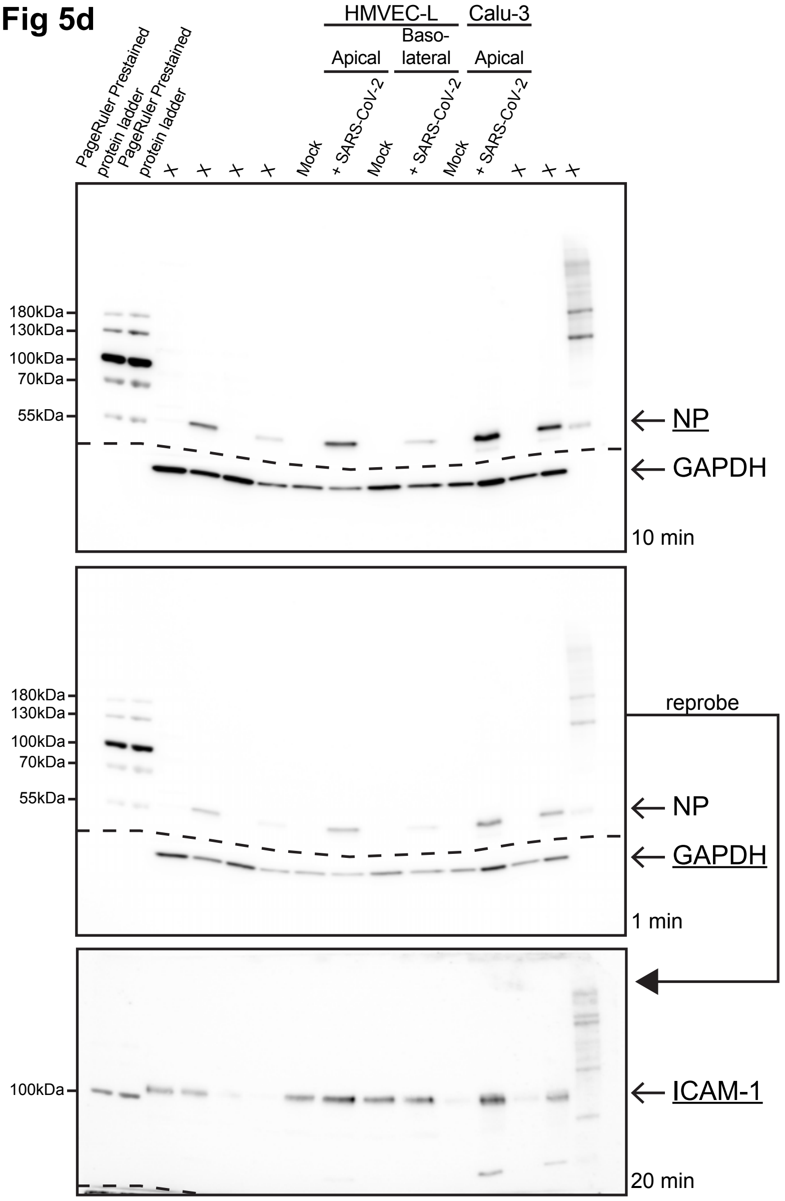

Supplementary figure 6

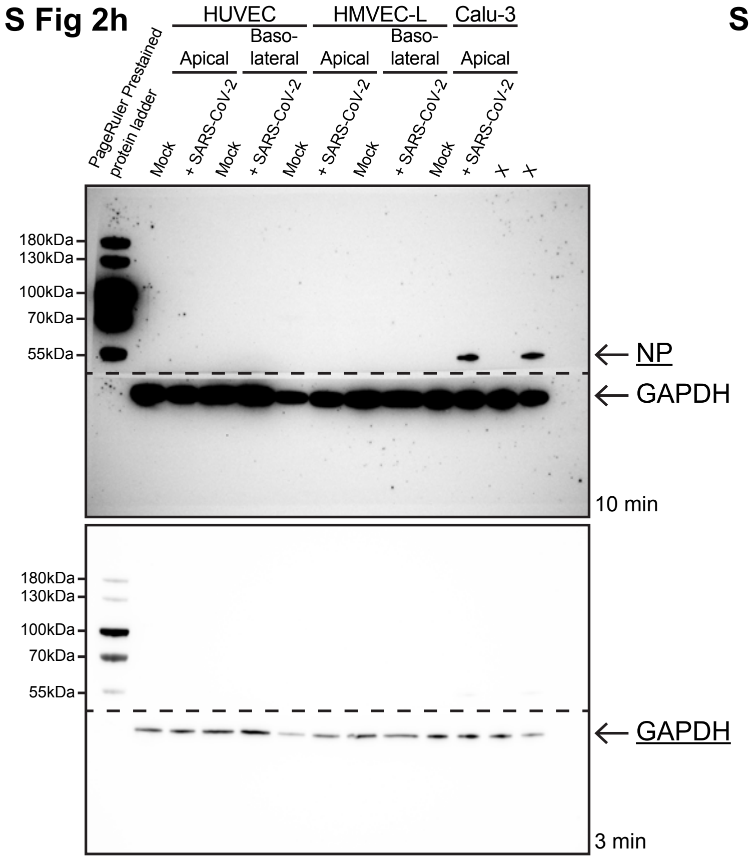

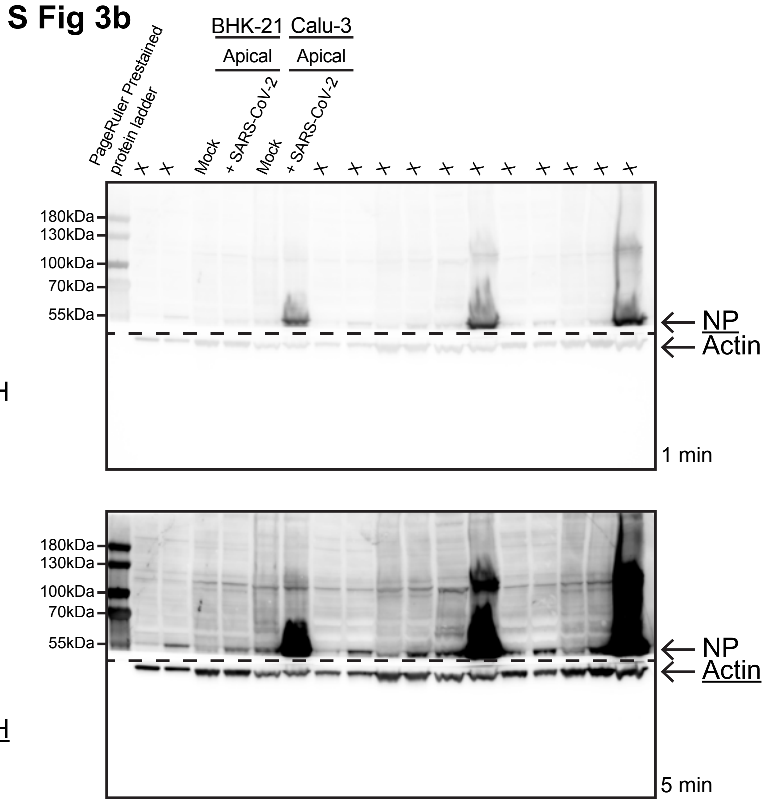

Supplementary figure 6. Full scans of Western blots. Full scans of Western blots shown in the paper figures. Used lanes are labelled as in the corresponding figure, with other lanes labelled as X. Dashed line indicates where membrane was cut. Underlined proteins indicate which blot was used in the corresponding figure and time indicates exposure time.
